# Co-administration of 20(S)-protopanaxatriol (g-PPT) and EGFR-TKI overcomes EGFR-TKI resistance by decreasing SCD1 induced lipid accumulation in non-small cell lung cancer

**DOI:** 10.1186/s13046-019-1120-4

**Published:** 2019-03-15

**Authors:** Quanfu Huang, Qiuguo Wang, Dong Li, Xiao Wei, Yijuan Jia, Zheng Zhang, Bo Ai, Xiaonian Cao, Tao Guo, Yongde Liao

**Affiliations:** 1 Department of Thoracic Surgery, Tongji Hospital, Tongji Medical College, Huazhong University of Science and Technology, Wuhan, Hubei 430030 People’s Republic of China; 2 Institute of Hematology, Union Hospital, Tongji Medical College, Huazhong University of Science and Technology, Wuhan, Hubei 430022 People’s Republic of China; 3 Cancer Biology Research Center (Key Laboratory of the Ministry of Education), Tongji Hospital, Tongji Medical College, Huazhong University of Science and Technology, Wuhan, Hubei 430030 People’s Republic of China; 4 Department of Obstetrics and Gynecology, Wuhan NO.1 Hospital, Wuhan, Hubei 430022 People’s Republic of China; 5 Department of Thoracic Surgery, Affiliated Yantai Yuhuangding Hospital of Qingdao University, Yantai, Shandong Province 264000 People’s Republic of China; 6 Department of Thoracic Surgery, Union Hospital, Tongji Medical College, Huazhong University of Science and Technology, Wuhan, Hubei 430022 People’s Republic of China; 7 Collaborative Innovation Center of Hematology, Huazhong University of Science and Technology, Wuhan, Hubei 430022 People’s Republic of China

**Keywords:** NSCLC, EGFR-TKI resistance, Lipid metabolism, Lipid droplet, SCD1, Oleic acid

## Abstract

**Background:**

Non-small cell lung cancer (NSCLC) patients with sensitive epidermal growth factor receptor (EGFR) mutations are successfully treated with EGFR tyrosine kinase inhibitors (EGFR-TKIs); however, resistance to treatment inevitably occurs. Given lipid metabolic reprogramming is widely known as a hallmark of cancer and intimately linked with EGFR-stimulated cancer growth. Activation of EGFR signal pathway increased monounsaturated fatty acids (MUFA) and lipid metabolism key enzyme Stearoyl-CoA Desaturase 1 (SCD1) expression. However the correlation between EGFR-TKI resistance and lipid metabolism remains to be determined.

**Methods:**

In this study the differences in lipid synthesis between paired TKI-sensitive and TKI-resistant patient tissues and NSCLC cell lines were explored. Oleic acid (OA, a kind of MUFA, the SCD1 enzymatic product) was used to simulate a high lipid metabolic environment and detected the affection on the cytotoxic effect of TKIs (Gefitinib and osimertinib) in cell lines with EGFR-activating mutations. (20S)-Protopanaxatriol (g-PPT), an aglycone of ginsenosides, has been reported to be an effective lipid metabolism inhibitor, was used to inhibit lipid metabolism. Additionally, synergism in cytotoxic effects and signal pathway activation were evaluated using CCK-8 assays, Western blotting, flow cytometry, Edu assays, plate clone formation assays and immunofluorescence. Furthermore, two xenograft mouse models were used to verify the in vitro results.

**Results:**

Gefitinib-resistant cells have higher lipid droplet content and SCD1 expression than Gefitinib-sensitive cells in both NSCLC cell lines and patient tissues. Additionally oleic acid (OA, a kind of MUFA, the SCD1 enzymatic product) abrogates the cytotoxic effect of both Gefitinib and osimertinib in cell lines with EGFR-activating mutations. As a reported effective lipid metabolism inhibitor, g-PPT significantly inhibited the expression of SCD1 in lung adenocarcinoma cells, and then down-regulated the content of intracellular lipid droplets. Combined treatment with Gefitinib and g-PPT reverses the resistance to Gefitinib and inhibits the activation of p-EGFR and the downstream signaling pathways.

**Conclusions:**

Our findings uncover a link between lipid metabolic reprogramming and EGFR-TKI resistance, confirmed that combination target both EGFR and abnormal lipid metabolism maybe a promising therapy for EGFR-TKI resistance and highlighting the possibility of monitoring lipid accumulation in tumors for predicting drug resistance.

**Electronic supplementary material:**

The online version of this article (10.1186/s13046-019-1120-4) contains supplementary material, which is available to authorized users.

## Introduction

Lung cancer is one of the most predominant and fatal cancers worldwide, and non-small cell lung cancer (NSCLC) represents approximately 85% of all lung cancer cases [1]. An important milestone in the treatment of NSCLC was the discovery of epidermal growth factor receptor (EGFR)-activating mutations as an effective therapeutic target and the successful development of third-generation EGFR tyrosine kinase inhibitors (EGFR-TKIs; Gefitinib, erlotinib, afatinib, and osimertinib). EGFR-TKIs act as competitive reversible inhibitors and provide significant clinical benefit to patients. However, the development of acquired resistance to these agents limits their long-term efficacy [2, 3], thereby necessitating the use of new therapeutic approaches or alternative strategies. More and more resistant mechanisms have been revealed in recent years, and lipid metabolic reprogramming maybe one of them. Alterations in lipid metabolism are now gaining recognition as a hallmark of cancer [4, 5]. The reprogramming of lipid metabolic pathways in cancer cells has been shown to play an important role in supporting cancer cell proliferation and survival [6, 7]. The dependence of tumor cells on deregulated metabolism/biosynthesis indicates that proteins involved in these processes may be attractive chemotherapeutic targets [8, 9].

As a key enzyme in lipid metabolism, Stearoyl-CoA Desaturase 1 (SCD1) synthesized saturated fatty acids (SFAs) into monounsaturated fatty acids (MUFAs), which can be further synthesized to neutral lipids such as triglycerides (TG) and stored in organelles termed ‘lipid droplets’ (LDs), and several studies have shown that cancer tissues display a higher rate of lipid droplets than normal tissues in several cancers including NSCLC [10–12]. Previous studies have demonstrated SCD1 is highly expressed and highlighted the involvement of SCD1 in the survival of NSCLC [13, 14].

Pisanu ME et al. reported that the use of combination therapy with SCD1 inhibitors reverts resistance to cisplatin in lung cancer stem cells, which highlight the role of SCD1 in cisplatin resistant [15]. It was also proved that in H460 lung cancer cells, the suppression of Stearoyl-CoA Desaturase (SCD) activity, impairs the ligand-induced phosphorylation of EGFR. Recently, evaluation of non-small cell lung cancer samples reveals a positive correlation among EGFR activation, SCD1 Y55 phosphorylation and SCD1 protein expression [16].

Even though a growing number of studies have demonstrated that EGFR-stimulated cancer growth depends on SCD1 activity especially in lung adenocarcinoma, no investigations have been carried out to identify the relevance of SCD1 expression linked to EGFR-TKI resistance in lung adenocarcinoma. Furthermore, the potential synergy between TKI therapy and SCD1 inhibition in lung adenocarcinoma has not yet been addressed. In this study, we investigated the relationship between SCD1 induced lipid synthesis and the mechanism of resistance to EGFR-TKIs in NSCLC. Our results confirmed an accumulation of intracellular lipid droplets (LDs) and higher SCD1 expression after resistance to EGFR-TKIs. Our study also proved that in EGFR-TKI-sensitive cell lines, OA (the SCD1 enzymatic product) abrogates the effect of TKIs (both first and third generation TKIs). (20S)-Protopanaxatriol (g-PPT) is an aglycone of ginsenosides isolated from *Panax ginseng* that has several interesting activities, including anticancer and antimetabolic effects [17, 18]. Given that our previous study confirm the lipid metabolism inhibition effect. As an extension of our previous research. Pharmacological treatment with g-PPT and Gefitinib triggers cell death in Gefitinib-resistant NSCLC cell lines. Furthermore, two xenografts were employed to confirm a hyposensitization to Gefitinib by OA and the reversal of Gefitinib resistance by the combination of g-PPT and Gefitinib Altogether, these results demonstrate that abnormal LD accumulation, SCD1 and lipid metabolism are candidate therapeutic targets for the treatment of TKI-resistant EGFR-mutant NSCLC and highlight the importance of detecting lipid metabolism in tumors to predict the emergence of EGFR-TKI resistance.

## Materials and methods

### Patients and samples

A total of 20 formalin-fixed paraffin-embedded tissue samples and frozen tissue samples were included in this study. These samples were obtained from 13 lung cancer patients (shown in Table 1). Case number 01–07 patients were diagnosed with primary NSCLC with cTNM stages of IIIB or IV and were unfit for surgery. Biopsy and EGFR mutational testing verified the presence of EGFR-TKI-sensitive mutations (ADx-ARMS, AmoyDx, China). After at least 2 months, first-generation EGFR-TKI (Gefitinib, AstraZeneca, UK) treatment (Patient’s medication time is up to 12 months and the shortest is 3 months) and clinical assessment according to the Response Evaluation Criteria In Solid Tumors (RECIST) confirmed cTNM downstaging to IIIA. The patients underwent initial surgery at the Department of Thoracic Surgery, Affiliated Tongji Hospital of Huazhong University of Science and Technology Tongji Medical College (Wuhan, China) from 2016 to 2018. Those patients harbor paired tissue of pre- and post- treatment. Case number 07–10 patients were underwent initial surgery after downstaging post-TKI treatment. For they initially subjected to EGFR mutational testing using peripheral blood, tissue samples were collected only after TKI treatment. Case number 11–13 underwent initial surgery at the Department of Thoracic Surgery during the same period and were confirmed to possess sensitive EGFR mutations.

**Table 1:** The baseline characteristics of the patients

Case No	Gender	Before TKI treatment			TKI treatment time (month)	After TKI treatment		
		Tumor Stage UICC	Tumor Stage TNM	EGFR mutation subtype		Tumor Stage UICC	Tumor Stage TNM	EGFR mutation subtype
01	Female	IV	cT2aN2M1	L858R	12	IIIA	pT2N2M0	T790 M/L858R
02	Female	IV	cT2aN2M1	19-Del	9	IB	pT2N0M0	T790 M
03	Female	IV	cT4N2M1	19-Del	7	IIIA	pT2N2M0	19-Del
04	Male	IV	cT1cN2M1	L858R	7	IA	pT1bN0M0	L858R
05	Male	IIIB	cT4N2M0	19-Del	3	IA	pT1N0M0	19-Del
06	Male	IIIC	cT3N3M0	19-Del	3	IB	pT2aN0M0	19-Del
07	Female	IIIA	cT2bN3M0	19-Del	2	IA	pT1N0M0	19-Del
08	Female	IIIB	cT3N3M0	19-Del	5	IB	pT2bN1M0	19-Del
09	Male	IIIA	cT2aN2M0	19-Del	3	IB	pT2aN0M0	19-Del
10	Male	IIIB	cT4N2M0	19-Del	3	IA	pT1N0M0	19-Del
11	Male	IB	pT2BN0M0	19-Del	N			
12	Male	IIIA	pT2N2M0	19-Del	N			
13	Female	IIB	pT3N0M0	L858R	N			

Case number 01–07 patients after biopsy and EGFR mutational testing verified the presence of EGFR-TKI-sensitive mutations, under treatment with TKIs. Finally confirmed cTNM downstaging to IIIA at least and then the patients underwent initial surgery. Those patients harbor paired tissue of pre- and post- treatment

Case number 07–10 patients were initially subjected to EGFR mutational testing using peripheral blood, tissue samples were collected only after TKI treatment. Case number 11–13 underwent initial surgery at the Department of Thoracic Surgery during the same period and were confirmed to possess sensitive EGFR mutations

All tissue samples including both paraffin-embedded tissue and frozen tissue were obtained with informed consent from patients. Each specimen has a corresponding tumor and adjacent tissues. None of the patients had any prior history of chemotherapy, radiation, or hormonal therapy except TKI treatment before the surgery. Clinical information of the patients was recorded in detail, and the diagnoses were confirmed by at least two pathologists. pTNM stage and tumor differentiation grade were obtained from the Tongji Hospital records. The baseline characteristics of the patients are shown in Table 1 and Additional file 1: Table S1. This study was approved by the Research Ethics Committee, Tongji Medical College, Huazhong University of Science and Technology. (The IRB ID number is TJ-IRB20180403).

### Cell lines and cell culture

The EGFR-mutant NSCLC cell lines HCC827, PC-9, and H1975 were originally obtained from ATCC and the Chinese Academy of Sciences (Shanghai, China) prior to 2014. Gefitinib-resistant HCC827GR (T790 M) cells were provided by Dr. J Yang (Guangxi Medical University, Guangxi, China) in 2016. All cell lines were cultured in RPMI 1640 medium (Gibco, Grand Island, NY, USA) supplemented with 10% fetal bovine serum (FBS; Gibco) and antibiotics (100 units/ml penicillin and 100 μg/ml streptomycin). All cell lines were cultured at 37 °C in a humidified atmosphere with 5% CO2 and 95% air.

### Cell treatment and cell morphological observation

Gefitinib (ZD1839) and osimertinib (AZD9291) were either supplied by AstraZeneca (Cambridge, UK) or purchased from MedChemExpress (MCE) (HY-50895/HY-15772, USA). g-PPT was purchased from MCE (HY-N0835, USA). OA was purchased from Sigma Chemical Co. (O1008, Sigma-Aldrich, USA). Cell culture experiments were performed using reagents formulated in 100% dimethyl sulfoxide (DMSO).

Equal amount of cells planted in a six-well plate and treated for 48 h. Cell were cultured at 37 °C in a humidified atmosphere with 5% CO2 and 95% air. After 48 h treatment the cell morphology and cell death were visualized with Inverted phase contrast microscope (Olympus, Tokyo, Japan).

### Oil red O staining

To detect the difference in basal LD content between the two groups of patients who received EGFR-TKI treatment or not, we performed Oil Red O staining. Frozen cancer tissues were embedded in OCT compound (Sakura, Tokyo, Japan) and cut into 10 μm sections. The sections washed several times with distilled water, followed by preincubation in 60% isopropanol prior to the final staining with filtered Oil Red O working solution (60% Oil Red O stock solution (BA-4081, Baso, Zhuhai, China) and 40% deionized water). After a series of washing steps in 60% isopropanol, the nuclei were counterstained with hematoxylin prior to differentiation in 1% hydrochloric acid in alcohol. Finally, after washing several times with distilled water, the slides were sealed with glycerin-gelatin. Representative images were captured using an inverted microscope (Olympus, Tokyo, Japan).

### Nile red staining

Live cells seeded on cover glasses were fixed in 4% paraformaldehyde (PFA) for 20 min at room temperature (RT) and then incubated with Nile red (HY-D0718, MCE, USA) at 1:2000 in phosphate-buffered saline (PBS) for 10 min. The slides were counterstained with Hoechst 33342 (H1399, Thermo Fisher, USA) at 1 μg/ml in PBS for 5 min at RT before imaging. The cells were visualized with a fluorescence microscope (Olympus, Tokyo, Japan). A representative image is shown from three independent experiments.

Fluorescence emitted by cells stained by Nile red was measured at 595 nm by flow cytometry (BD Biosciences, USA), during the assay 10,000 cells per sample were collect and count. The result analyzed with Cell Quest software (BD Biosciences, USA) and expressed as mean of fluorescence intensity (MFI).

### Immunohistochemistry (IHC)

Formalin-fixed paraffin-embedded tissue blocks were retrieved from the archive and analyzed by IHC as previously described [13, 19]. In short, the Avidin-Biotin Complex (ABC) Vectastain Kit (ZSGB-Bio, Beijing, China) was used, and anti-SCD1 (ab19862), anti-p-EGFR1068 (#3777), anti-c-Caspase3 (#9664), anti-KI67 (ab15580), and anti-perilipin (#9349) were used as primary antibodies to incubate the tissue sections (4-μm thick) after heat-induced epitope retrieval (in 10 mM sodium citrate buffer of pH 6.0), followed by incubation with a secondary antibody conjugated to peroxidase (1:100; Dako). Detection was performed using diaminobenzidine for 3 min, and the slides were counterstained with hematoxylin. The scoring system used incorporated both the intensity of scoring (0 = absent, −, 1 = weak, +, 2 = moderate, ++, and 3 = strong, +++) and the percentage of positive tumor cells (0 = 0%, 1 = 1–25%, 2 = 26–50%, 3 = 51–75%, and 4 = 76–100%). Points for the intensity and percentage of staining were added to calculate the overall score according to the method described before [20]. Investigators scored all slides for SCD1, perilipin, KI67, c-Caspase3 and p-EGFR expression and followed the criteria of double-blind trials. Based on the overall score, SCD1, perilipin, KI67, c-Caspase3 and p-EGFR expression was classified as negative (≤ 4) and positive (> 4).

### Cell viability assay

Eight thousand cells per well were transferred to 96-well flat-bottomed plates and cultured overnight before exposure to various concentrations of Gefitinib, osimertinib, and OA in medium containing 10% FBS for 48 h. After incubation for the indicated times, cell proliferation was measured. In brief, 10 μl commercial Cell Counting Kit-8 (CCK-8, Dojindo Molecular Technologies, Inc., Japan) reagent was added to each well and incubated at 37 °C for 1 h. Absorbance was measured at 450 nm using a spectrophotometer. Each experiment was performed in triplicate and repeated at least 3 times.

### Western blotting of cultured cells

Cells were harvested and lysed using ice-cold RIPA lysis buffer (50 mM Tris-HCl (pH 7.4), 150 mM sodium chloride, 1% Nonidet P-40, and 0.5% sodium deoxycholate) supplemented with a protease inhibitor cocktail (Roche). Following centrifugation at 10,000×g for 15 min at 4 °C, proteins in the supernatants were quantified by the Bradford method and separated using 10% SDS-PAGE gels and electrotransferred from the gel to nitrocellulose membranes (Merck & Co., Inc., Whitehouse Station, NJ, USA). Following blocking with 5% skimmed milk in PBS, the membranes cut into strips according to the different molecular weights. The membranes were immunoblotted with the primary antibodies against p-ERK (#4370), (#4695), AKT (#4691), p-AKT (#4060), c-PARP (#5625), c-Caspase3 (#9664), p-EGFR1068 (#3777) and p-Stat3 (#9145), BCL-XL (10783–1-AP), GAPDH (10494–1-AP), KI67 (ab15580), c-MET (ab51067), and SCD1 (ab19862) at 4 °C overnight. Subsequently, HRP-conjugated secondary antibodies bound to the primary antibodies were detected using an ECL detection system (ChemiDocTM XRS+ machine, Bio-Rad Laboratories). GAPDH protein levels were employed as loading controls. Densitometric analyses were performed using ImageJ software. Relative quantification was carried out after normalization against the band intensities of GAPDH. A Mann-Whitney test was performed to assess the difference in protein expression between groups. A representative blot is shown from three independent experiments. The expression of target protein by image lab (Bio-Rad Laboratories).

### Apoptosis analysis using flow cytometry

An Annexin V-FITC apoptosis kit (BD Biosciences, NJ, USA) was used to determine the number of apoptotic cells according to the manufacturer’s instructions. Different cell groups were harvested with 0.25% trypsin and washed with PBS. After centrifugation, the cells were re-suspended in 100 μl buffer and then stained with Annexin V (3 μl) and propidium iodide (PI; 5 μl), and the mixture was incubated in the dark at 4 °C for 15 min. The cells were sorted using a FACS Calibur flow cytometer (BD Biosciences, USA), during the assay 10,000 cells per sample were collect and count. The result were analyzed using Cell Quest software (BD Biosciences, USA). The experiments were repeated three times.

### BODIPY 493/503 staining

Cells were fixed in 4% PFA for 20 min at RT and incubated with BODIPY 493/503 (D3299, Thermo Fisher, USA) at 1:2000 and Hoechst 33342 (H1399, Thermo Fisher, USA) in PBS for 15 min at RT. Finally, the cells were visualized with a fluorescence microscope (Olympus, Tokyo, Japan). A representative image is shown from three independent experiments.

For LD staining, live cells were washed twice in PBS and incubated in 2 μg/mL BODIPY in PBS for 15 min at 37 °C. After staining, cells were washed twice in PBS and fixed in 2% paraformaldehyde for 15 min. Fixed cells were washed and re-suspended in PBS, passed through a cell strainer, and analyzed on an FACS Calibur flow cytometer (BD Biosciences, USA) under FL-1.

### Immunofluorescence (IF) staining of cultured cells and mouse lung cancer sections

Cells cultured in cover slides were pretreated with drugs for 48 h. The cells were fixed with 4% PFA for 20 min, permeabilized with 0.1% Triton™ X-100 (30–5140 SAJ, Sigma-Aldrich, USA) for 10 min, blocked with 5% bovine serum albumin (BSA) for 1 h and incubated with antibodies against p-EGFR 1068 and SCD1 (1:100, rabbit) at 4 °C overnight. Highly cross-adsorbed donkey anti-rabbit IgG (H + L) secondary antibody was used at a concentration of 2 μg/ml in PBS containing 0.2% BSA for 1 h at RT to label the cells, and then the cells were counterstained with 1 μg/ml Hoechst 33342 for 5 min at RT. The cells were visualized with a fluorescence microscope (Olympus, Tokyo, Japan).

Mouse lung tumors were prepared and subjected to IHC staining as described above. The Avidin-Biotin Complex (ABC) Vectastain Kit (ZSGB-Bio, Beijing, China) was used, and anti-p-EGFR 1068 and anti-SCD1 (1:100, rabbit) were used as primary antibodies to incubate the tissue sections (4-μm thick) after heat-induced epitope retrieval (with 10 mM sodium citrate buffer of pH 6.0) and blockage with 5% BSA for 1 h. Highly cross-adsorbed donkey anti-rabbit IgG (H + L) secondary antibody was used at a concentration of 2 μg/ml in PBS containing 0.2% BSA for 1 h at RT to label the cells, and then the cells were counterstained with Hoechst 33342 (H1399, Thermo Fisher, USA). The tissues were visualized with a fluorescence microscope (Olympus, Tokyo, Japan). The fluorescence intensity Quantified by image J (Rawak Software, Inc. Germany).

### Cell proliferation assay and plate clone formation assay

After 48 h of treatment, cell proliferation was quantified based on the incorporation of 5-ethynyl-2′-deoxyuridine (Edu) into DNA using a Cell-Light™ Edu Apollo®567 In Vitro Imaging Kit (Rio-Bio, Guangzhou, China). Before fixation, the cells were incubated with Edu for 2 h, permeabilized in 1.0% Triton X-100 for 15 min, and then subjected to Edu staining. Cell nuclei were stained with Hoechst 33342 (H1399, Thermo Fisher, USA) for 15 min. Fluorescence microscopy (Olympus, Tokyo, Japan) was used to determine the proportion of nucleated cells that had incorporated Edu. The cell proliferation rate was calculated as a percentage of Edu-positive nuclei to total nuclei in five high-power fields per well. The assay was performed in triplicate and repeated three times in independent experiments.

The plate clone formation assay was performed using the indicated cells. For each group, 800 surviving cells per well were incubated in 6-well plates containing 2 ml complete medium per well, followed by incubation for 10 days. Then, treatment was commenced when a mass of cells was visible to the naked eye for 48 h. At the indicated time point, the cells were washed twice with PBS, treated with crystal violet for 10 min, washed again, counted, and assessed. All experiments were performed at least 3 times. Colonies with > 40 cells were counted under phase contrast microscopy at × 40 magnification.

### Xenograft mouse models

Four-week-old female Kunming mice (Animal Purchase No. 11401300077528, No. 32002100004219) were obtained from the Experimental Animal Center of Hubei Province (Animal Study Permit No. SCXK 2010–0009) and maintained in an environment with a standardized barrier system (System Barrier Environment No. 00021082) in the Experimental Animal Center of Tongji Hospital of Huazhong University of Science and Technology. These manipulations were approved by the Animal Care and Use Committee in Tongji Hospital of Huazhong University of Science and Technology. To establish the H1975-luc xenograft model, which is resistant to Gefitinib, and the HCC827-luc xenograft model, which is sensitive to Gefitinib, we transfected H1975 and HCC827 cells with luciferase and confirmed the efficiency of transfection using the IVIS Spectrum system (Caliper Life Sciences Inc., Xenogen Corporation).

Approximately 1 × 10^7^ H1975-luc cells were subcutaneously injected into the right hind limbs of mice. Treatment began 1 week following injection. The mice were randomized into four groups (*n* = 4 per group) and intraperitoneally injected with vehicle (PBS, NT), g-PPT (50 mg/kg/day), Gefitinib (50 mg/kg/day) or g-PPT (50 mg/kg/day) + Gefitinib (50 mg/kg/day).

In a parallel experiment, 1 × 10^7^ HCC827-luc cells were subcutaneously injected into the left upper limbs of mice. Treatment began 1 week following injection. The mice were randomized into three groups and intraperitoneally injected with vehicle (PBS, NT), Gefitinib (50 mg/kg/day) or Gefitinib (50 mg/kg/day) + OA (50 mg/kg/day) (*n* = 4 per group).

Tumor growth was monitored using caliper measurements twice every week, and tumor volume was calculated using the formula length x width^2^ × 0.52. Body weight was assessed twice weekly. Approximately 4 weeks later, the mice were analyzed using the IVIS Spectrum system (Caliper Life Sciences Inc., Xenogen Corporation). The total flux (photons/s) of the xenografts was calculated using Living Image software version 4.3.1. Then, the xenografts from each group were collected for further IHC and IF analyses.

### Bioinformatics and public database analyses

Gene expression data (GSE83666, GSE38310 profiling data) were downloaded as raw signals from the Gene Expression Omnibus (http://www.ncbi.nlm.nih.gov/geo), interpreted, normalized and log2-scaled using the online analysis tool GCBI website (https://www.gcbi.com.cn). The gene expression of SCD1, perilipin (PLIN) and FASN in NSCLC cell lines was obtained from published gene expression profiles included in the Cancer Genome Atlas (TCGA) dataset using cBioPortal tools (http://cbioportal.org).

### Statistics

The results are expressed as the mean ± standard deviation (SD) from at least three independent experiments. Single comparisons between two groups were performed by Student’s t-tests. Comparisons between multiple groups were performed by one-way ANOVA followed by Tukey’s post-test. All statistical analyses were performed in SPSS 18.0.0 (SPSS Inc., Chicago, IL). *p* values < 0.05 were considered significant.

## Results

### SCD1 expression and lipid droplet accumulation increase after EGFR-TKI treatment or TKI resistance occur

**Fig. 1:**
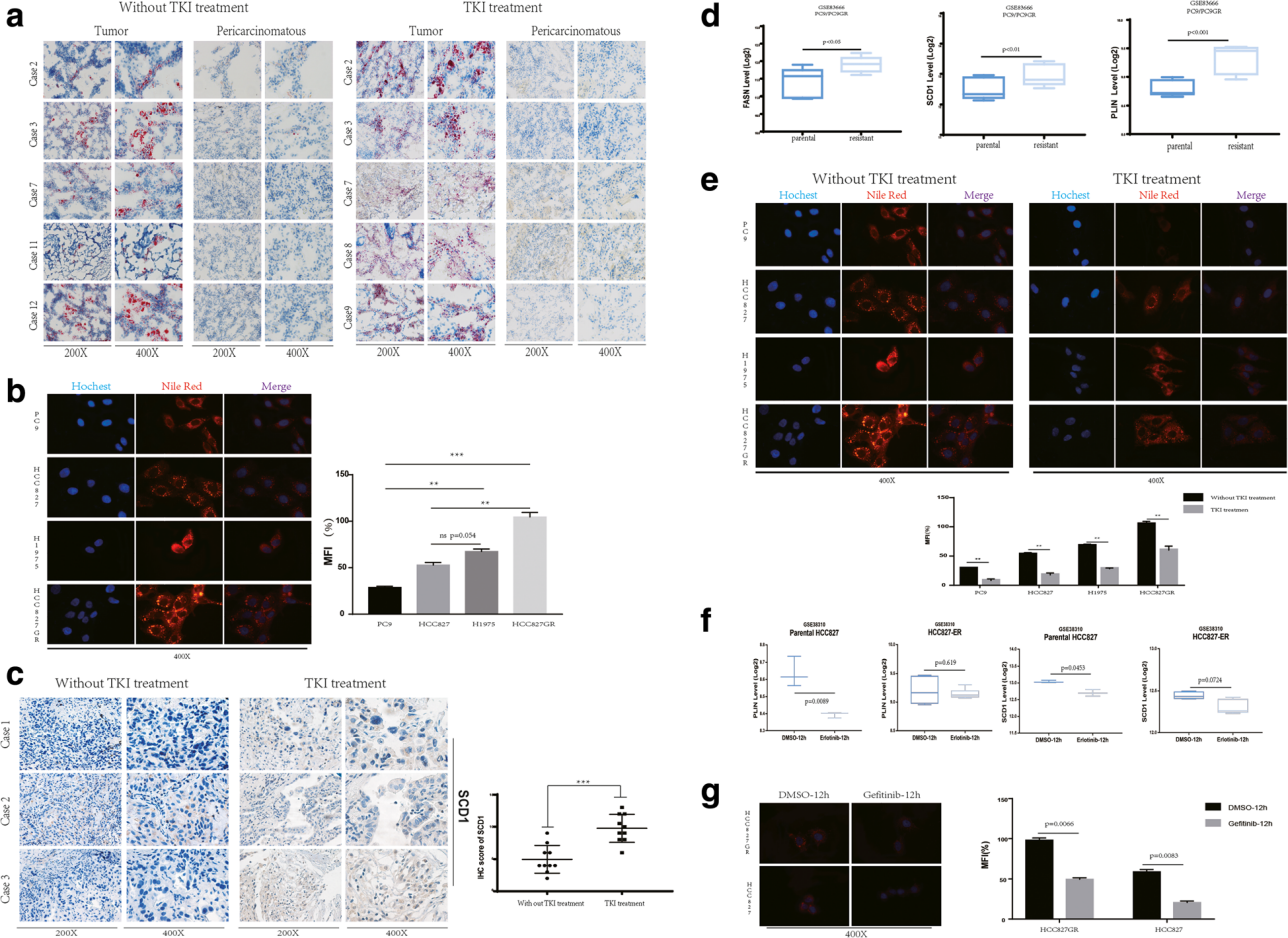
LD accumulation and fatty acid metabolism increase during EGFR-TKI treatment (**a**) Basal LD content of both tumors and adjacent tissues were assessed by Oil Red O staining between two groups of patients who received EGFR-TKI treatment and patients who did not. **b** Left panel, basal LD content of different mutation status cell lines assessed by Nile red staining. Right panel, quantitation of Nile red staining of each cell lines. Lipid accumulation was evaluated by measuring Nile red fluorescence by flow cytometry; data are expressed as the percentage of level found in control cells treated only by the vehicle (HCC827GR) and are the means ± SD of at least 3 independent experiments. *p < 0.05, **p < 0.01, when compared to control cells. **c** Left panel, immunohistochemical staining for SCD1 protein in paired patient tumor tissues before and after TKI treatment. Brown color in cancer cells denotes positive staining. Representative images of SCD1 expression in NSCLC specimens from three representative patients are shown. Right panel, the immunohistochemical score for SCD1 was significantly different between the pre- and post-Gefitinib treatment specimens. *p* values were determined by unpaired t test. ***p < 0.001. **d** Boxplots showing the expression level of SCD1/FASN/PLIN in the GSE83666 dataset. **e** Up panel, basal LD content assessed by Nile red staining after 48 h of treatment with Gefitinib. Down panel, quantitation of Nile red staining of each cell lines. The quantitative method is the same as above, the vehicle is (HCC827GR without treatment) **f** Boxplots showing the expression level of PLIN/SCD1 in HCC827/HCC827ER after 12 h exposed in Erlotinib in the microdissected of GSE38310. **g** Left panel, basal LD content assessed by Nile red staining after 12 h of treatment with Gefitinib. Right panel, quantitation of Nile red staining by flow cytometry; the quantitative method is the same as above, and the vehicle is (HCC827GR DMSO-12 h).

In our study, we used pre- and post-TKI treatment specimens, including matched tissues and contemporaneous surgical specimens shown in Table 1. We first evaluated and compared the basal LD content of the specimens pre- and post-TKI treatment by Oil Red O staining. A significant difference was observed between tumor and pericancer tissues. Only the tumor tissues were stained by Oil Red O, and nearly no staining was observed in the pericancer tissues. Meanwhile, the specimens from patients who underwent TKI treatment displayed higher Oil Red O staining than the specimens from patients who did not (Fig. 1a). We next investigated whether the NSCLC cell lines displayed a similar trend. To this end, the cell lines with sensitive EGFR mutations PC9 (19-Del) and HCC827 (L858R), the cell line with mutations associated with primary resistance to EGFR-TKIs H1975 (L858R/T790 M), the cell line with mutations associated with acquired resistance to EGFR-TKIs HCC827-GR (Gefitinib-resistant, T790 M) were stained with Nile red. When we stained the cell lines with Nile red to explore whether lipid droplets expression associated with cell line mutations status. As shown in Fig. 1b, the extent of Nile red staining of HCC827GR significantly higher than its parental cell line HCC827 and PC9. Similar result observed in H1975, even though when compared with HCC827 show no statistical difference. The extent of Nile red staining was much higher in the cell lines with resistant EGFR mutations (including both cell line with acquired resistance (HCC827GR) and cell line with primary resistance (H1975)) than in the cell lines with sensitive EGFR mutations (Fig. 1b). All above, we found the lipid droplets accumulated after a long-term treatment with TKIs.

Given the fact the lipid droplets in NSCLC undergoes significant changes before and after Gefitinib treatment. We further detected whether the expression of some key protein of lipid metabolic have any change during the treatment of Gefitinib. SCD1 is a key enzyme in lipid metabolism and is involved in the introduction of a double bond into palmitic and stearic acids, giving rise to palmitoleic acid and OA, respectively [21]. Perilipin family members (PLIN1–5), the proteins surrounding LDs, are regarded as the most important regulators of LDs, facilitating LD movement and interaction among cellular signaling components [22]. To determine whether SCD1/PLIN expression changed during Gefitinib treatment, we investigated the expression of SCD1/PLIN by IHC. Paired tumor tissues from patients both before and after at least 3 months of Gefitinib treatment were used. The expression of SCD1/PLIN was upregulated after Gefitinib treatment (Fig. 1c; Additional file 2: Figure S1a). Immunohistochemical scoring of SCD1 expression showed a statistically significant difference between the pre- and post-Gefitinib treatment specimens (Fig. 1c; Additional file 2: Figure S1b, *p* < 0.01). Analysis of the public database GSE83666 also proved that the resistant cell lines PC9GR higher express the lipid metabolism associated gene FASN, SCD1 and PLIN compared with the parental cell lines PC9 which quite consistent with the part of cell lines staining with Nile red (Fig. 1d; *p* < 0.05, p < 0.01, *p* < 0.001 respectively). Taken together, our data confirm the accumulation of LDs and lipid metabolic associated gene upregulated connection with EGFR-TKI resistance.

In view of increased lipid droplets and lipid metabolic associated gene after long-term TKIs treatment. We further investigated the expression of LDs in a short time treatment by staining the cell lines with Nile red after exposing them to Gefitinib for 48 h. Interestingly, the staining intensity was reduced compared with that in cells without Gefitinib exposure, which quite different with the situation of long-term treatment. When we further measured the difference in each cell line with or without Gefitinib treatment, we found that the cell lines with sensitive EGFR mutation displayed a greater decrease than the other cell lines (Fig. 1e). Profiles (GSE38310) of parental HCC827 and TKIs resistant cell line HCC827ER after exposed to TKIs 12 h was analyzed to support this view. As shown in Fig. 1f the lipid metabolic associated gene SCD1/PLIN decreased after 12 h treatment, and compared with TKI-resistant cells (HCC827ER), the level of SCD1/PLIN decline was significantly higher in the parental cells (HCC827) after 12 h of treatment. The Nile red staining of both HCC827GR and parental HCC827 after 12 h Gefitinib treatment also show a decrease of staining intensity (Fig. 1g). Both the result of treatment for 12 h or 48 h fit in with the above part of the public database profiles analysis. When a short-term treatment with TKIs, NSCLC cell decrease the lipid droplets expression to response Gefitinib short term treatment in both HCC827 and HCC827GR.

Therefore, we can conclude that NSCLC cells decrease LD content in response to short-term Gefitinib treatment, however intracellular LDs of post-treatment patient tissue and HCC827GR accumulate after long-term Gefitinib treatment compared with per-treatment tissue and parental HCC827. Additionally, the key enzyme SCD1 and the LD-surrounding proteins perilipins are upregulated after Gefitinib treatment in NSCLC, indicating that abnormal lipid metabolism may be responsible for Gefitinib resistance.

### Supplement of oleic acid decreases the sensitivity of cell lines with sensitive EGFR mutations to EGFR-TKIs

In a recent study researcher found EGFR stabilizes SCD1 and up-regulating MUFA synthesis to promote lung cancer growth. Our study confirmed NSCLC cells decrease LD content and lipid metabolic associated gene in response to short-term Gefitinib treatment. And finally an accumulation of LDs and increased lipid metabolic associated gene PLIN and SCD1 expression in the Gefitinib-resistant cell lines and tissues during a long term gefitinib treatment. Therefore, we further explore whether there exists a mechanism or factor that can lead to hyposensitivity to TKIs. OA is an abundant MUFA that results from lipid metabolism, and SCD1 plays a key role during this process [23]. In recent studies, the function of OA in promoting cancer metastasis has been increasingly accepted [24].

**Fig. 2:**
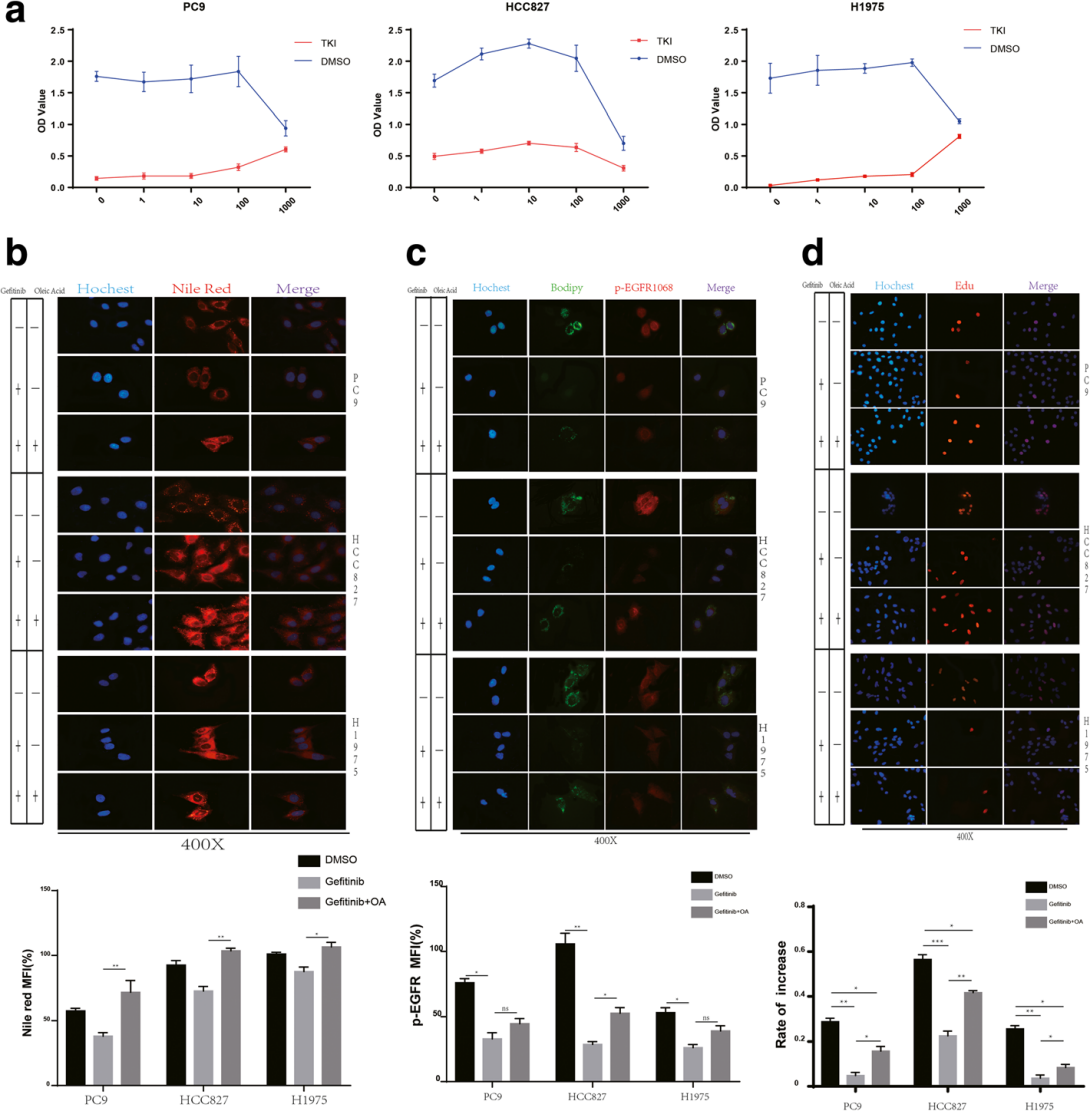
Hyposensitivity to EGFR-TKI after the treatment of EGFR-sensitive NSCLC cell lines with OA (**a**) The indicated NSCLC cell lines were exposed to varying concentrations (0, 1 μM, 10 μM, 100 μM, and 1000 μM) of OA with vehicle (DMSO, NT) or Gefitinib (15 μM or 20 μM in H1975 cells) for 2 days, and the relative OD value was assessed to determine cell viability by the CCK-8 assay. Data represent the mean ± SD of three replicate determinations. **b** Up panel, the cells were stained with Nile red 48 h after vehicle (DMSO, NT), Gefitinib (100 nM or 20 μM in H1975 cells), or Gefitinib (100 nM) + OA (100 μM) treatment. Down panel, lipid accumulation of indicated cell lines was evaluated by measuring Nile red fluorescence by flow cytometry; data are expressed as the percentage of level found in control cells (H1975 DMSO) and are the means ± SD of at least 3 independent experiments. *p < 0.05, **p < 0.01, when compared to control cells. **c** Up panel, HCC827, PC9 and H1975 cells were stained with BODIPY 493/503 (green) and anti-p-EGFR1086 antibody (red) and counterstained with Hoechst (blue) after 48 h of exposure to vehicle (DMSO, NT), Gefitinib (100 nM or 20 μM in H1975 cells), or Gefitinib (100 nM) + OA (100 μM). Down panel, quantitation of p-EGFR expression of indicated cell lines. **d** Up panel, Edu staining was used to quantify the inhibition of cell proliferation. The indicated NSCLC cell lines were seeded and exposed to vehicle (DMSO, NT), Gefitinib (100 nM or 20 μM in H1975 cells), or Gefitinib (100 nM) + OA (100 μM) for 48 h and stained for Edu. Down panel, the proliferation rate was determined by counting the proliferating cells using ImageJ software. *p* values were determined by one-way ANOVA. *p < 0.05, **p < 0.01, ***p < 0.001. Data represent the mean ± SD of three replicate determinations

**Fig. 3:**
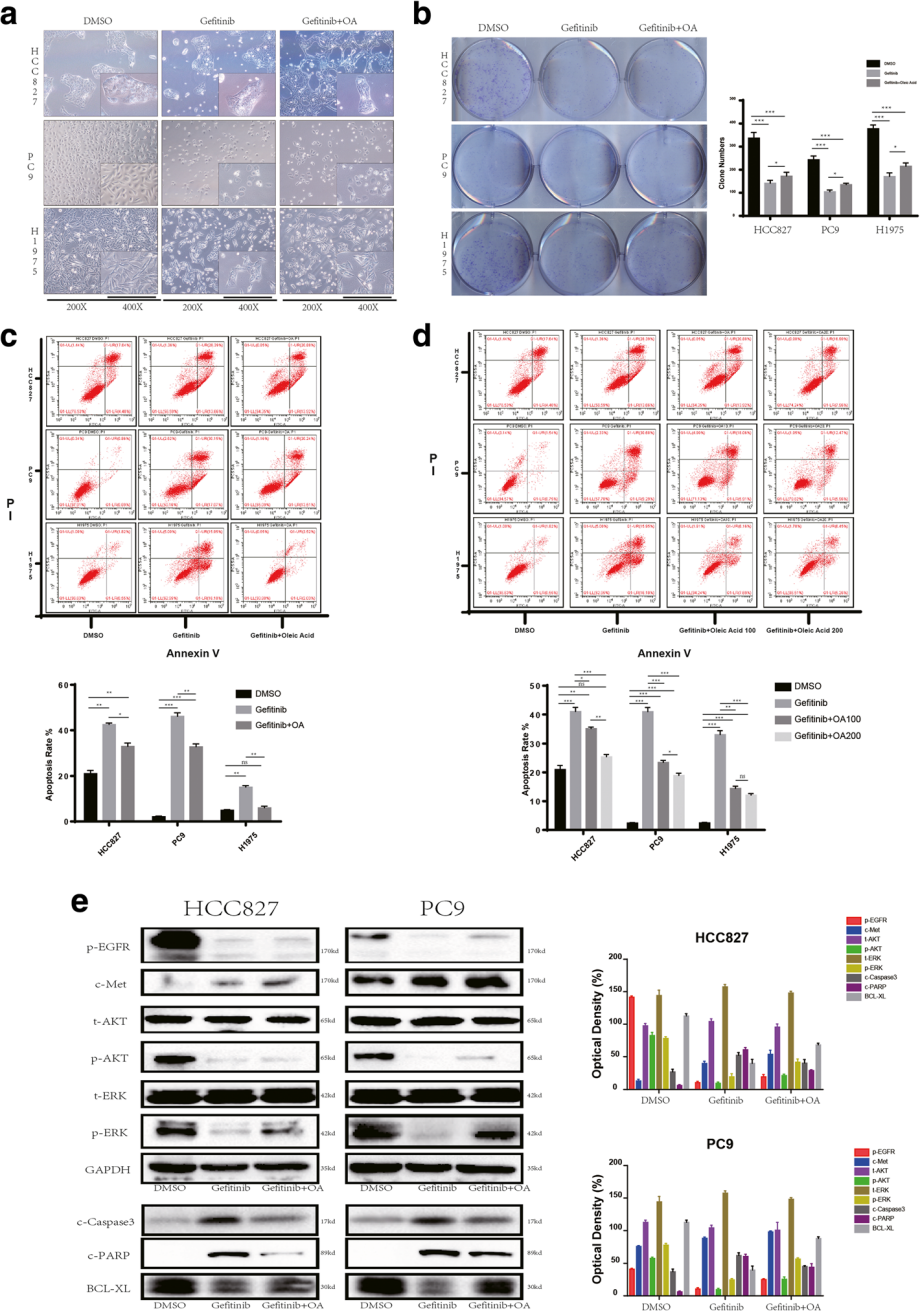
OA abrogates the cytotoxic effect of EGFR-TKIs on EGFR-sensitive NSCLC cell lines (**a**) Morphology of the indicated NSCLC cell lines after vehicle (DMSO, NT), Gefitinib (100 nM or 20 μM in H1975 cells), or Gefitinib (100 nM) + OA (100 μM) exposure was assessed. For each experiment, five images of random fields were acquired. A representative image is shown. **b** Left panel, 800 surviving cells per well were seeded in 6-well plates, followed by incubation for 10 days until a mass of cells was visible to the naked eye. The synchronized cell masses were exposed to vehicle, Gefitinib, or Gefitinib + OA for 48 h and stained with crystal violet. Right panel, the number of colonies was assessed using ImageJ software. *p* values were determined by one-way ANOVA. *p < 0.05, **p < 0.01, ***p < 0.001. Data represent the mean ± SD of three replicate determinations. **(c)** Upper panel, apoptotic rates of the indicated NSCLC cell lines were assessed by flow cytometry after vehicle, Gefitinib, or Gefitinib + OA treatment for 48 h. Lower panel, apoptotic rates were quantified. The results represent data from three independent experiments. p values were determined by one-way ANOVA. *p < 0.05, **p < 0.01, ***p < 0.001. **d** Upper panel, apoptotic rates of the indicated NSCLC cell lines were assessed by flow cytometry after vehicle (DMSO, NT), Gefitinib (100 nM or 20 μM in H1975 cells), Gefitinib (100 nM) + OA (100 μM), or Gefitinib (100 nM) + OA (200 μM) treatment for 48 h. Lower panel, apoptotic rates were quantified. The results represent data from three independent experiments. *p* values were determined by one-way ANOVA. *p < 0.05, **p < 0.01, ***p < 0.001. **e** The indicated cell lines were exposed to vehicle, Gefitinib, or Gefitinib + OA for 48 h. The cells were then harvested for Western blotting to detect the indicated signaling proteins (upper panel) and apoptotic proteins (lower panel). A representative blot is shown from three independent experiments. Right panel, the quantitation of western bolt

To test whether OA could decrease sensitivity to Gefitinib in the cell lines with sensitive EGFR mutations HCC827 and PC9, we exposed the cells to various concentrations of OA with or without Gefitinib treatment and assessed proliferation by CCK-8 assays. Within a certain concentration range, OA increased cell proliferation both with and without Gefitinib administration (Fig. 2a). We further investigated the desensitization effect of OA in response to treatment with osimertinib, a third-generation TKI. Similarly, co-administration of osimertinib with OA resulted in hyposensitivity to EGFR-TKIs (Additional file 3: Figure S2a). Nile red staining was performed to detect changes in LD content. Treatment with Gefitinib decreased LD content; however, co-treatment of the indicated cell lines with Gefitinib and OA resulted in a higher LD content than treatment with Gefitinib alone (Fig. 2b). Next, cell proliferation based on Edu staining and p-EGFR expression was assessed. The IF results showed that compared to Gefitinib treatment alone, co-administration of Gefitinib with OA increased the expression of p-EGFR especially in HCC827 cell line, meanwhile in PC9 and H1975 also witness an increased the expression of p-EGFR affection even thorough no statists difference (Fig. 2c). Additionally, co-treatment with OA and Gefitinib resulted in greater Edu staining and a higher proliferation rate than did Gefitinib treatment alone (Fig. 2d, *p* < 0.05). To clarify the mechanism of the TKI hyposensitivity effect induced by OA, we further investigated the expression of signaling pathway proteins. Gefitinib significantly inhibited the activation of p-EGFR/p-AKT/p-ERK; however, the activation of p-EGFR/p-AKT/p-ERK was recovered when Gefitinib was co-administered with OA (Fig. 3e).

After 48 h treatment the Gefitinib and OA co-treatment group was characterized by a reduction in morphological changes and cell death compared with Gefitinib single treatment group (Fig. 3a). The plate clone formation assay also showed a similar result, i.e., the group co-administered with OA and Gefitinib had more colonies than the group administered with Gefitinib alone (Fig. 3b). The apoptosis rates assayed by flow cytometry and apoptosis-associated protein expression were used to evaluate the extent of cytotoxicity to determine the effect of OA in reducing Gefitinib-induced apoptosis. Indeed, the expression of apoptosis indexes c-PARP and c-Caspase3 was decreased, and the expression of the antiapoptosis index BCL-XL was increased in HCC827 and PC9 cells in response to OA (Fig. 3e). In accordance with Western blotting results, the apoptosis rates decreased after OA treatment (Fig. 3c, *p* < 0.05). Additionally, within a certain concentration range, the effect of OA decreased with increasing concentrations (Fig. 3d, *p* < 0.05). We also observed similar results when OA was co-administered with osimertinib (Additional file 3: Figure S2b).

### In vivo verification of the Gefitinib hyposensitivity effect induced by oleic acid

**Fig. 4:**
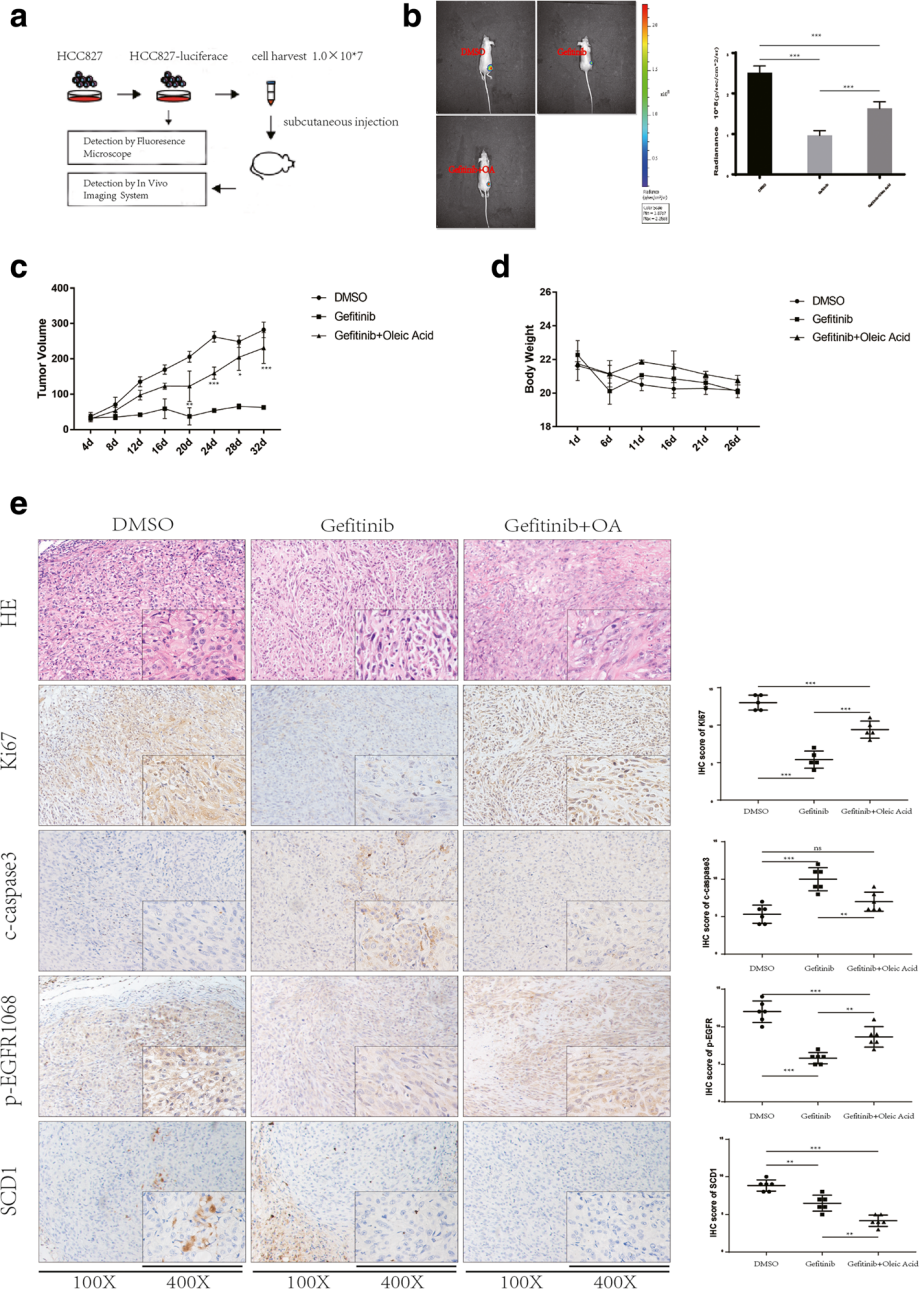
Combined treatment with OA and EGFR-TKIs abrogates the cytotoxic effect of EGFR-TKIs in vivo a Flowchart of the in vivo experiments. **b** In vivo luciferase activity measured after the establishment of tumor in mice pretreated for four weeks with vehicle (PBS, NT), Gefitinib (5 mg/kg/day) or Gefitinib (5 mg/kg/day) + OA (5 mg/kg/day). Quantification of luminescence is represented as the radiance. **c-d** Tumor weights and Tumor growth curves for each group were obtained. *p* values were determined by one-way ANOVA. *p < 0.05, **p < 0.01, ***p < 0.001. Data represent the mean ± SD of three replicate determinations. **e** HE and IHC staining for KI67, c-Caspase3, p-EGFR and SCD1. Data represent the mean ± SD of three replicate determinations. p values were determined by one-way ANOVA. *p < 0.05, **p < 0.01, ***p < 0.001. Error bars denote the SD

To test whether OA could induce hyposensitivity to Gefitinib and reduce Gefitinib-induced apoptosis in Gefitinib-sensitive NSCLC cells in vivo, we evaluated the combined effect of OA and Gefitinib using a HCC827 murine xenograft tumor model (Fig. 4a). Treatment began 1 week following injection. The mice were randomized into three groups and intraperitoneally injected with vehicle (PBS, NT), Gefitinib (5 mg/kg/day) or OA (5 mg/kg/day) + Gefitinib (5 mg/kg/day). After measuring the tumors for the last time, we analyzed the mice in each group using the IVIS Spectrum system and sacrificed them. Tumor masses were isolated and analyzed by IHC. Treatment with Gefitinib or Gefitinib + OA inhibited tumor progression compared to treatment with the vehicle, while treatment with Gefitinib alone significantly suppressed tumor growth compared to treatment with Gefitinib + OA (Fig. 4b, c). Negligible reduction was observed in the body weight of mice in all groups (Fig. 4d). Consistent with the in vitro results, combined OA and Gefitinib treatment resulted in noticeably higher cytotoxic effect of Gefitinib and led to higher p-EGFR/KI67 expression and lower c-Caspase3 expression than treatment with Gefitinib alone (*p* < 0.05) (Fig. 4e). Combined treatment with OA and Gefitinib also inhibited SCD1 expression, as OA is a downstream product of LD metabolism driven by SCD1. Furthermore, IF for p-EGFR expression was performed on these tumor masses, and in accordance with the IHC results, a recovery in p-EGFR activation was observed upon the co-administration of Gefitinib with OA (Additional file 4: Figure S3).

### Combined treatment with g-PPT and Gefitinib decreases intracellular LD content by inhibiting SCD1 expression

**Fig. 5:**
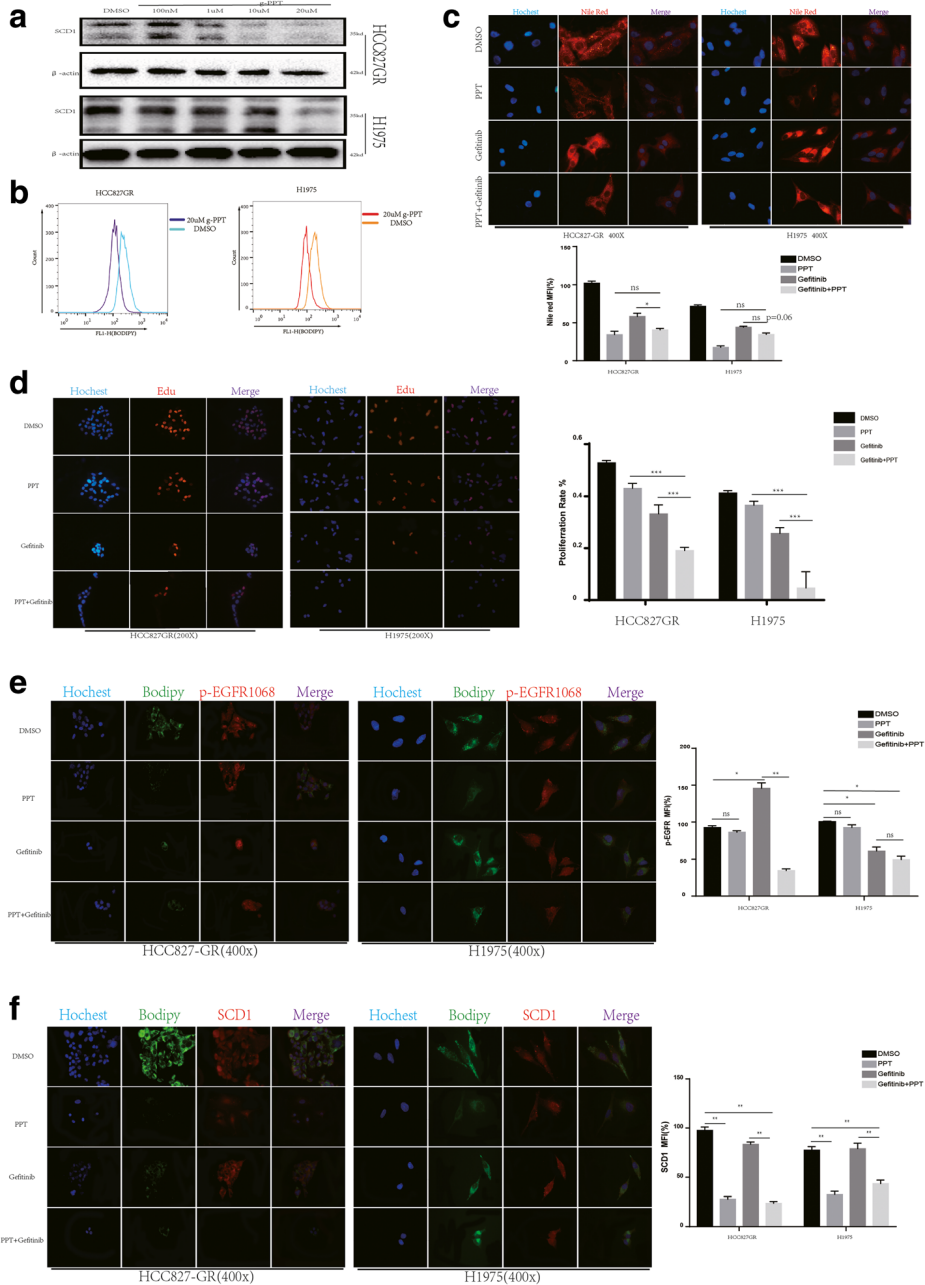
Combined treatment with g-PPT and Gefitinib decreases intracellular LD content by inhibiting SCD1 expression (**a**) The indicated NSCLC cell lines were exposed to concentrated depend g-PPT (100 nM, 1 μM, 10 μM, 20 μM) for 48 h and then harvested for Western blotting to detect the indicated signaling proteins. A representative blot is shown from three independent experiments. **b** Cell lines were seeded and exposed to vehicle (DMSO, NT), g-PPT (20 μM) for 48 h and then incubated in BODIPY for flow cytometer analysis. **c** Up panel, the indicated NSCLC cell lines were seeded and exposed to vehicle (DMSO, NT), g-PPT (20 μM), Gefitinib (2 μM) or g-PPT (20 μM) + Gefitinib (2 μM) for 48 h, and LD content was assessed by Nile red staining. Down panel, quantitation of lipid accumulation of indicated cell lines was evaluated by measuring Nile red fluorescence by flow cytometry; data are expressed as the percentage of level found in control cells treated only by the vehicle (HCC827GR DMSO) and are the means ± SD of at least 3 independent experiments. *p < 0.05, **p < 0.01, when compared to control cells. **d** Left panel, Edu staining was used to quantify the inhibition of cell proliferation. Cell lines were exposed to vehicle, g-PPT, Gefitinib or g-PPT + Gefitinib for 48 h and stained for Edu. Right panel, the proliferation rate was determined by counting the proliferating cells using ImageJ software. *p* values were determined by one-way ANOVA. *p < 0.05, **p < 0.01, ***p < 0.001. **e** Left panel, the indicated cell lines were exposed to vehicle, g-PPT, Gefitinib or g-PPT + Gefitinib for 48 h, and the content of LDs and expression p-EGFR1068 were assessed by IF. Right panel, quantitation of p-EGFR expression of indicated cell lines. **f** Left panel, the given cell lines after exposed for 48 h and assessed the expression of lipid droplets and SCD1 by immunofluorescence. Right panel, quantitation of SCD1 expression of indicated cell lines. *p* value calculated as above.

Given that an accumulation of LDs/SCD1 expression may be responsible for Gefitinib resistance and administrate with OA lead to induce LD expression and hyposensitivity to TKIs, we wondered whether combined targeting of EGFR and abnormal lipid metabolism can reverse resistance to Gefitinib in the indicated NSCLC cell lines. To this end, we used a concentrated dependent manner g-PPT to inhibit lipid metabolism, the expression of SCD1 were detected by western blot and BODIPY staining to detect lipid droplets by flow cytometry. Both In HCC827GR and H1975 cell lines, treatment with concentrated g-PPT resulted in SCD1 expression decreased (Fig. 5a). Meanwhile the BODIPY staining to track the content of LDs by flow cytometry also show a lipid metabolism inhibition effect of g-PPT (Fig. 5b).

To further investigate whether combination therapy with lipid metabolism inhibitors to achieve better control of lung cancer TKIs resistance, Gefitinib-resistant cell lines HCC827GR and H1975 were treatment with Gefitinib and g-PPT single used or in combination. Nile red staining was performed to detect the changes in intracellular LDs. In response to g-PPT, regardless of the single or combination treatment, intracellular LD content was markedly decreased in HCC827GR, same effect observe in H1975 cell line even through the decreased without statists difference (Fig. 5c), which in match with BODIPY staining indicating that g-PPT indeed inhibits intracellular LD accumulation. As is known to all abnormal activation of the EGFR signal is an important cause of lung cancer cell proliferation. To further explore the effect of combination therapy on NSCLC cell proliferation and the subsequent changes in the expression of p-EGFR and SCD1 caused by the inhibition of LD accumulation, we performed Edu and IF assays. In HCC827GR and H1975 cell lines, combination treatment with g-PPT resulted in fewer cells with Edu staining and a sharp decrease in the proliferation rate (Fig. 5d, *p* < 0.05). The expression of p-EGFR, which is an important indicator of cell proliferation, was also significantly lower in the combined treatment group than in the Gefitinib alone treatment group in the HCC827GR cell line. A same effect observe in H1975 cell line even through the decrease without statists difference. We also performed BODIPY staining to track the content of LDs (Fig. 5e). After g-PPT treatment, the expression of SCD1 was inhibited in both the combination treatment group and the single treatment group, whereas p-EGFR was inhibited only when g-PPT was combined with Gefitinib (Fig. 5f). Therefore, the combination treatment decreased both the accumulation of LDs and the expression of SCD1 and p-EGFR, thereby inhibiting cell proliferation.

### Combination therapy inhibits EGFR- ERK pathway activation and induces the apoptosis of Gefitinib-resistant cell

**Fig. 6:**
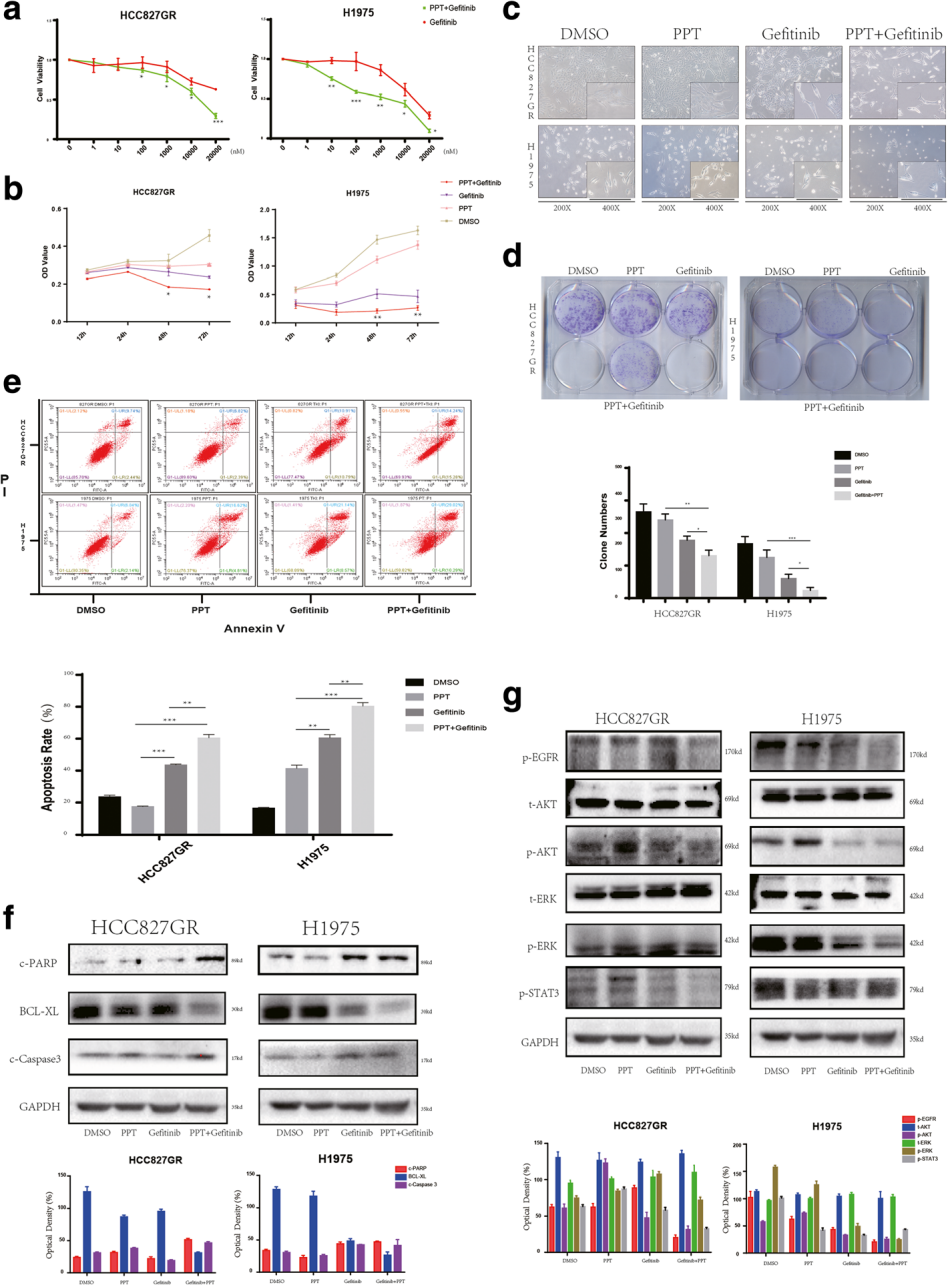
Effect of combined treatment with g-PPT and Gefitinib on apoptosis in acquired resistant cell lines **a** The indicated NSCLC cell lines were exposed to various concentrations of Gefitinib and Gefitinib + g-PPT (20 μM) for 2 days. Cell proliferation was determined by the CCK-8 assay. Data represent the mean ± SD of three replicate determinations. **b** Relative OD value at 12, 24, 48 and 72 h after vehicle (DMSO, NT), g-PPT (20 μM), Gefitinib (2 μM) or g-PPT (20 μM) + Gefitinib (2 μM) treatment. Data are expressed as cell viability from three independent experiments. **c** Morphology of the indicated NSCLC cell lines after exposure to vehicle, g-PPT, Gefitinib or g-PPT + Gefitinib was assessed. For each experiment, five images of random fields were acquired. A representative image is shown. **d** Upper panel, 800 surviving cells per well were incubated in 6-well plates, followed by incubation for 10 days until a mass of cells was visible to the naked eye. The synchronized cell masses were exposed to vehicle, g-PPT, Gefitinib or g-PPT + Gefitinib for 48 h and stained with crystal violet. Lower panel, the colony numbers were assessed with ImageJ software. p values were determined by one-way ANOVA. *p < 0.05, **p < 0.01, ***p < 0.001. **e** Upper panel, apoptotic rate of the indicated NSCLC cell lines were assessed by flow cytometry after vehicle, g-PPT, Gefitinib or g-PPT + Gefitinib treatment for 48 h. Down panel, apoptotic rates were quantified. *p* values calculated as above. **f** Up panel, cell lines were exposed to vehicle, g-PPT, Gefitinib or g-PPT + Gefitinib for 48 h. The cells were harvested for Western blotting to detect the indicated apoptotic proteins. A representative blot is shown from three independent experiments. Down panel, the quantitation of western bolt. **g** Up panel, western bolt detect intracellular signal change in TKI-resistant cell lines. The cells were then harvested for Western blotting to detect the indicated signaling proteins. A representative blot is shown from three independent experiments. Down panel, the quantitation of western bolt

Given that an accumulation of LDs and lipid metabolism may be responsible for Gefitinib resistance, combination therapy with g-PPT inhibit LDs accumulation and down-regulated SCD1 expression. We wondered whether combined targeting of EGFR and abnormal lipid metabolism can reverse resistance to Gefitinib in the indicated NSCLC cell lines. To this end, we compared the combination of g-PPT (20 μM) and Gefitinib (2 μM) with Gefitinib (2 μM) only at various concentrations for 48 h. The CCK-8 assay was performed to detect the cytotoxic effect. As shown in Fig. 6a, significant differences were observed with the combined treatment with g-PPT. The combination treatment group was more sensitive to Gefitinib than the Gefitinib-only treatment group. To further determine whether such a phenomenon occurs in a time-dependent manner, the indicated cell lines were exposed to vehicle (DMSO, NT), g-PPT (20 μM), Gefitinib (2 μM) or g-PPT (20 μM) + Gefitinib (2 μM), and cell proliferation was determined by the CCK-8 assay at 12, 24, 48 and 72 h after treatment. Combination therapy group also displayed a lower OD value in a time-dependent manner than the other groups (Fig. 6b). We also found that the cell morphology was substantially different among the groups. The combination therapy groups were characterized by a greater extent of morphological changes and more dead cells (Fig. 6c). To further investigate the effect of combination treatment, we performed a special plate clone formation assay. To this end, synchronized cells were incubated in 6-well plates for 10 days and exposed to vehicle (DMSO, NT), g-PPT (20 μM), Gefitinib (2 μM) or g-PPT (20 μM) + Gefitinib (2 μM) for 48 h, followed by staining with crystal violet and counting the number of colonies. Compared with the vehicle, the co-administration of Gefitinib and g-PPT drastically reduced the number of colonies, and in contrast with Gefitinib (*p* < 0.05) or g-PPT (*p* < 0.001) treatment alone, combined treatment with Gefitinib and g-PPT slightly but significantly decreased the number of colonies (Fig. 6d).

Gefitinib-induced apoptosis was also analyzed by flow cytometry. The number of apoptotic cells was used to assess cytotoxicity in the indicated EGFR-TKI-resistant cell lines. The numbers of apoptotic cells in the combination group were significantly higher than those in the vehicle and single drug treatment groups (Fig. 6e, *p* < 0.01). However, when we exposed the EGFR-TKI-sensitive cell lines to the same treatment, we obtained different results. HCC827 cells in the combination treatment group displayed a higher rate of apoptosis than cells in the single Gefitinib group, while PC9 cells did not (Additional file 5: Figure S4). Among all the groups of PC9 cells, the Gefitinib single treatment group had the highest rate of apoptosis; however, combined treatment with g-PPT weakened the apoptotic effect of Gefitinib. Subsequently, the Gefitinib-resistant cell lines HCC827GR and H1975 were exposed to vehicle (DMSO, NT), g-PPT (20 μM), Gefitinib (2 μM) or g-PPT (20 μM) + Gefitinib (2 μM) for 48 h and then harvested for Western blotting to detect the indicated apoptotic proteins. Apoptosis relate protein such as c-PARP and c-Caspase3 markedly increased, and the anti-apoptosis related protein BCL-XL sharply decreased in the Gefitinib-resistant cell line HCC827GR. In H1975 cells, despite displaying almost the same trend as HCC827GR cells, the apoptosis related protein did not change as significantly as in HCC827GR cells (Fig. 6f). In our opinion, this may be because of the special EGFR mutational status of H1975 cells, which possess simultaneous T790 M/L858R mutations.

To further clarify the antitumor effects of g-PPT in combination with Gefitinib, we examined the effects of this drug combination on signaling pathways in HCC827GR and H1975 cells (Fig. 6g). p-EGFR activity was inhibited by Gefitinib alone in H1975 cells but not in HCC827GR cells, likely because of for the mutational status of H1975 cells. In both cell lines, Gefitinib had a partial inhibitory effect on the activation of AKT, ERK and STAT3, which act downstream of EGFR; meanwhile, g-PPT hardly had any effect. However, co-administration of g-PPT with Gefitinib caused an apparent reduction in p-AKT, p-ERK and p-STAT3 levels. Simultaneous inhibition of p-AKT, p-ERK, p-STAT3 and p-EGFR was only achieved using the combination of Gefitinib and g-PPT.

Collectively, given the limited effect of g-PPT or Gefitinib alone in TKI-resistant cell lines, these data suggest that combined treatment with g-PPT and Gefitinib can synergistically enhance the apoptotic effect of Gefitinib and revert resistance to Gefitinib.

### In vivo antitumor activity of combined g-PPT and Gefitinib therapy

**Fig. 7:**
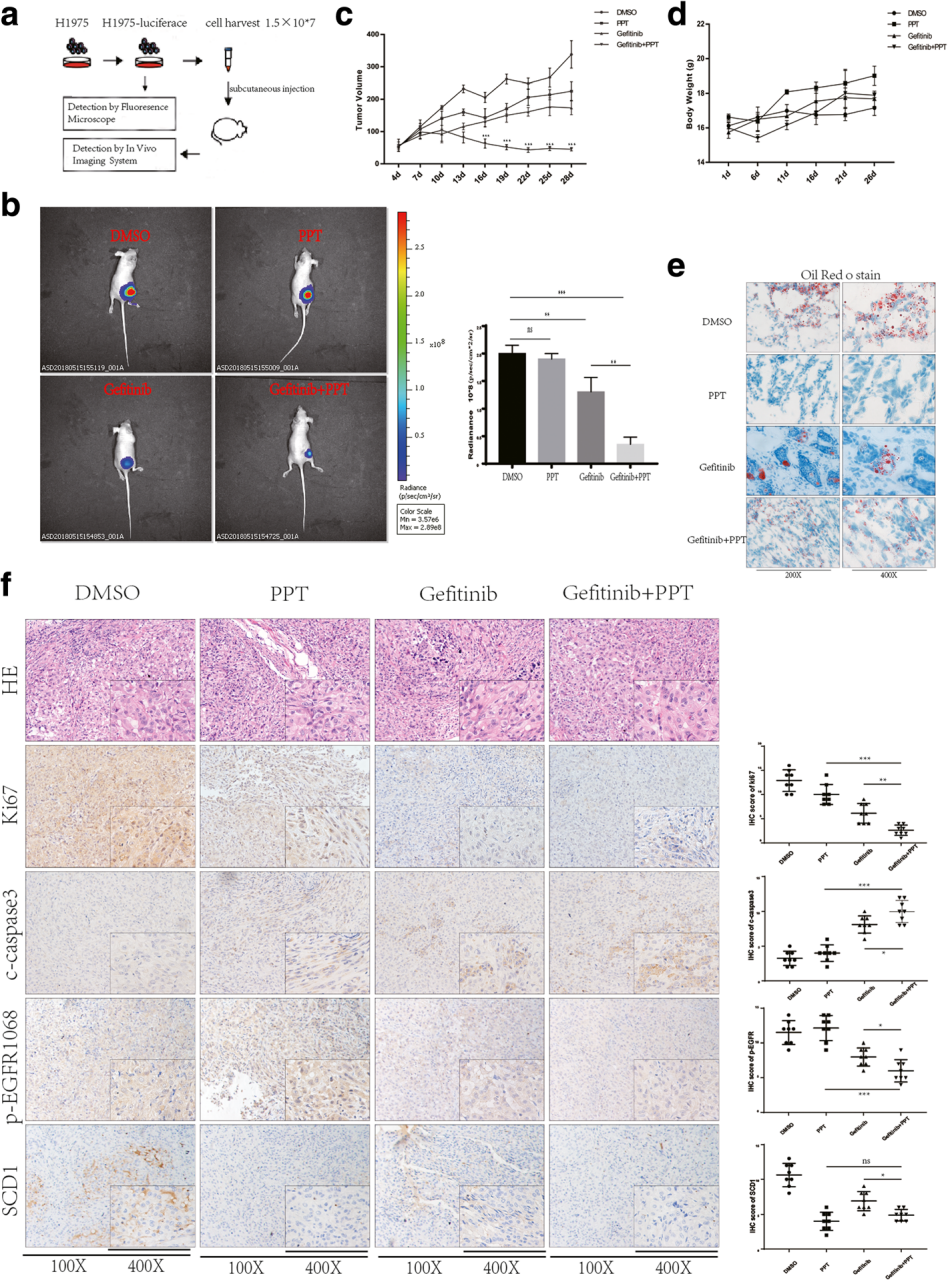
Efficacy of g-PPT combined with EGFR-TKIs in an H1975 tumor xenograft model (**a**) Flowchart of the in vivo experiments. **b** In vivo luciferase activity measured after the establishment of tumors in mice pretreated for four weeks with vehicle (PBS, NT), g-PPT (10 mg/kg/day), Gefitinib (50 mg/kg/day) or g-PPT (10 mg/kg/day) + Gefitinib (50 mg/kg/day). Quantification of luminescence is represented as the radiance. **c-d** Tumor weights and tumor growth curves for each group were obtained. p values were determined by one-way ANOVA. *p < 0.05, **p < 0.01, ***p < 0.001. Data represent the mean ± SD of three replicate determinations. **e** Basal LD content of four groups of frozen subcutaneous tumor slices was assessed by Oil Red O staining (× 200/× 400 magnification). A representative image is shown from three independent experiments. **f** HE and IHC staining for KI67, c-Caspase3, p-EGFR and SCD1. Data represent the mean ± SD of three replicate determinations. *p* values were determined by one-way ANOVA. *p < 0.05, **p < 0.01, ***p < 0.001. Error bars denote the SD

To test whether g-PPT could increase sensitivity to Gefitinib and enhance Gefitinib-induced apoptosis in Gefitinib-resistant NSCLC cells in vivo, we evaluated the combined effect of g-PPT and Gefitinib using a H1975 murine xenograft tumor model (Fig. 7a). Treatment began 1 week following injection. The mice were randomized into four groups and intraperitoneally injected with vehicle (PBS, NT), g-PPT (10 mg/kg/day), Gefitinib (50 mg/kg/day) or g-PPT (10 mg/kg/day) + Gefitinib (50 mg/kg/day). After measuring the tumor for the last time, we analyzed the mice in each group using the IVIS Spectrum system and sacrificed them. Tumors were isolated and analyzed by IHC. Tumors treated with PPT barely inhibit, Gefitinib alone treatment exhibited inhibited tumor progression relative to tumors treated with the vehicle, and treatment with the combination of the two agents significantly suppressed tumor growth (Fig. 7b, c). Additionally, the combination therapy was well tolerated, as evidenced by the negligible reduction in body weight compared with than with the other treatments (Fig. 7d). Consistent with the in vitro results, the combined g-PPT and Gefitinib treatment clearly reduced p-EGFR and KI67 expression and increased c-Caspase3 expression compared to Gefitinib or g-PPT treatment alone (Fig. 7f, *p* < 0.05). IF and Oil Red O staining were also performed on these tumor masses, and combination treatment with the two agents significantly suppressed p-EGFR expression and LD accumulation (Fig. 7e; Additional file 6: Figure S5). The in vivo results confirmed that g-PPT synergizes with Gefitinib to inhibit xenograft growth, consistent with the in vitro results.

## Discussion

The efficacy of conventional and molecular-targeted cancer therapies has been hindered by the emergence of drug resistance to therapy. Although EGFR-TKIs are the standard of care for patients with EGFR-mutant NSCLC, TKIs are not curative, mainly due to rapid progression after drug resistance [19, 25, 26]. Currently, several major resistance mechanisms to TKIs have been identified and characterized, including T790 M and C797S mutations and Met amplification [27–29]. The mechanism underlying the emergence of resistance is still unclear, and the origin of the resistant mutations remains controversial [30–32]. Hence, there is an unmet need to identify the mechanism underlying resistance and novel targets in NSCLC for therapeutic intervention.

Lipid metabolic reprogramming is widely known as a hallmark of cancer that enables cancer cells to adapt and sustain survival signals [33–35]. Previous joint drug trials have confirmed that blocking abnormal lipid metabolism can solve chemotherapy resistance or endocrine therapy resistance [15, 34]. Accumulating evidence suggests that EGFR mutations can drive alterations in metabolism [16, 36, 37], indicating that metabolic reprogramming plays a vital role in the emergence of EGFR-TKI resistance. To this end, our initial approach involved assessing the content of LDs and the expression of SCD1. SCD1 is known as a key enzyme in lipid metabolism that is involved in the introduction of a double bond into palmitic and stearic acids, giving rise to palmitoleic acid and oleic acid, respectively [38]. Paired pre- and post-TKI treatment tumor tissues were collected, and we observed LD accumulation and SCD1 expression upregulated in post-TKI treatment tissues. Cell lines with different sensitivities to TKIs also confirmed that the extent of LDs was much higher in the cell lines with resistant EGFR mutations (including both cell line with acquired resistance (HCC827GR) and cell line with primary resistance (H1975)) than in the cell lines with sensitive EGFR mutations. Analysis of paired TKI resistance cell line PC9GR and parental PC9 data from public databases also supported our findings and indicated a correlation between abnormal lipid metabolism and TKI resistance. In our study the post-TKI treatment tissue expression higher LD. Additionally resistance cell lines show higher LD expression and database analysis the resistance cell line PC9GR and parental PC9 cell line, PC9GR show higher lipid metabolic associated gene expression. Taken together we thought this may indicated lipid metabolism correlation to resistance. A recent report also suggested that FASN mediates resistance in NSCLC [39]. We also found that although the cell lines with different sensitivities to TKIs had variable LD content, when treated with TKI for a short time, the LD content decreased in all cell lines. We think that this is due to the adaptation of cells to sustain survival signals, and LDs are mobilized and metabolized into small molecule phospholipids and fatty acids to maintain the survival of cells. SCD1 can regulate the ratio between SFAs and MUFAs and affect membrane fluidity and cell function. Previous studies have confirmed the effect of altered plasma membrane fluidity on cell proliferation [40–42]. This led us to hypothesize that SCD1 may induce resistance in NSCLC by increasing membrane fluidity. Nevertheless, the definite mechanism underlying sustained NSCLC cell survival by increasing membrane fluidity requires further study.

One important observation of this study was that OA abrogated the cytotoxic effect of TKIs in cell lines with sensitive EGFR mutations. OA is a major product of intracellular lipid metabolism (MUFA) triggered by SCD1. Accumulating evidence suggests that OA alters the activation of signaling pathways to promote cancer progression [43, 44]. It known to all TKIs inhibit the phosphorylation of EGFR and the activation of the downstream pathway leads to cytotoxicity in EGFR sensitive mutation NSCLC cells; Zhang et al. reported that EGFR stabilizes SCD1 through Y55 phosphorylation and up-regulating MUFA synthesis to promote lung cancer growth [16]. SCD1 can be subtly controlled by tyrosine phosphorylation and uncover a previously unknown direct linkage between oncogenic receptor tyrosine kinase and lipid metabolism in lung cancer. In our study although the effects are different in different cell lines, the trend is similar. We found co-treatment with OA lead to an accumulation of LDs, abrogates the effect of EGFR-TKIs and recovers the proliferation ability of cells, finally sustaining cell survival both in vitro and in vivo. Our results corroborate previous findings, suggesting that high OA expression triggered by SCD1 acts as a central node connecting lipid metabolism with therapeutic resistance. Taken together, our data demonstrate that abnormal lipid metabolism may be responsible for resistance to EGFR-TKIs.

Given that LDs accumulate and SCD1 upregulated in TKIs resistant cell lines, OA lead to an accumulation of LDs and abrogated the cytotoxic effect of TKIs. To detect whether inhibit abnormal lipid metabolism can reverse the resistance of NSCLC cells to TKIs, we used g-PPT to inhibit lipid metabolism. g-PPT has been proved with several interesting activities, including anticancer and antimetabolic effects [17, 18, 45]. Whereas, a limited effect of g-PPT and TKI single used was observed in TKI-resistant NSCLC cell lines. However, when we co-treated TKI-resistant cells with g-PPT and Gefitinib, we observed a synergistic cytotoxic effect both in vitro and in vivo. Interestingly, this synergistic cytotoxic effect was not the same in TKI-resistant cells (HCC827GR/H1975) as in TKI-sensitive cells (HCC827/PC9), which may be due to differences in lipid metabolism between TKI-sensitive cells and TKI-resistant cells. There are also differences in primary resistant (the sensitive and resistant mixed) cell lines H1975 when compared with the secondary resistance cell line HCC827GR, which proves that the mutation state of the cells itself has a different effect on the combination therapy. Additionally, it is well known that ligand binding to EGFR phosphorylates EGFR, finally activating the downstream signaling pathways. Both the AKT and ERK signaling pathways are known to be downstream targets of EGFR, and EGFR activating mutations have been reported to deregulate these pathways in cancer [46, 47]. In our study the level p-EGFR and the activation of both ERK/AKT pathways of Gefitinib-resistance cell lines was sharply decreased in the combination treatment group, meanwhile Gefitinib not. This result suggests that co-administration of EGFR-TKIs with g-PPT inhibit the expression of SCD1 and affects EGFR phosphorylation which provide a linkage between EGFR-TKI resistance and lipid metabolism in lung cancer.

## Conclusions

EGFR-TKI resistance cannot be avoided, and the early detection of drug resistance and a timely change in treatment are the main ways to prolong survival after the emergence of drug resistance. Improvements in the understanding of tumor metabolism in recent years have demonstrated the necessity of complex metabolic rewiring in tumor cells for adaptations to adverse conditions for survival. Our results reveal a relationship between abnormal lipid metabolism and EGFR-TKI resistance for the first time. Synergistic treatment with g-PPT and TKIs can reverse the resistance to TKIs by targeting abnormal LD accumulation and SCD1 expression. These findings suggest that stratification of patients based on lipid metabolism could help optimize treatment plans and predict resistance to improve therapeutic outcomes in patients with activating EGFR mutations.

## Additional files

Additional file 1: **Table S1.** Specimen type of patient collected. (DOCX 17 kb)

Additional file 2: **Figure S1.** The difference of PLIN expression between the pre- and post-Gefitinib treatment specimens **(a)** Immunohistochemical staining for perilipin proteins in 20 lung cancer tissues from 10 patients. The brown color in cancer cells denotes positive staining. Representative images of perilipin expression in NSCLC specimens from three representative patients are shown, including samples from both before and after EGFR-TKI treatment (× 200/× 400 magnification). **(b)** The immunohistochemical score for PLIN was significantly different between the pre- and post-Gefitinib treatment specimens. *p* values were determined by unpaired t test. ***p < 0.001. (TIF 10243 kb)

Additional file 3: **Figure S2.** OA abrogates the cytotoxic effect of osimertinib in EGFR sensitive mutation cell lines **(a)** The indicated NSCLC cell lines were exposed to various concentrations (0, 0.5 nM, 5 nM, 50 nM, 500 nM, and 5 μM) of osimertinib with vehicle (DMSO, NT) or OA (100 μM) for 2 days, and the relative OD value, representing the cell viability by CCK-8 assays, was assessed. Data represent the mean ± SD of three replicate determinations. **(b)** Upper panel, apoptotic rates of the indicated NSCLC cell lines were assessed by flow cytometry after vehicle (DMSO, NT), osimertinib (150 nM) or osimertinib (150 nM) + OA (100 μM) treatment for 48 h. Lower panel, apoptotic rates were quantified. The results represent data from three independent experiments. p values were determined by one-way ANOVA. *p < 0.05, **p < 0.01, ***p < 0.001. (TIF 3020 kb)

Additional file 4: **Figure S3.** When treatment with OA, induce the expression of p-EGFR in murine tumors Up panel, IF was used to detect the expression of p-EGFR in murine lung tumors with an anti-p-EGFR1086 antibody (red, left panel) and counterstaining with Hoechst (blue). For each experiment, five images of random fields were acquired. A representative image is shown (× 200/× 400 magnification). Down panel, the quantitation of p-EGFR, p values were determined by one-way ANOVA. *p < 0.05, **p < 0.01, ***p < 0.001. Data represent the mean ± SD of three replicate determinations. (TIF 9912 kb)

Additional file 5: **Figure S4.** The combination treatment of Gefitinib and g-PPT in EGFR sensitive mutation NSCLC cell lines Upper panel, apoptotic rates of the indicated NSCLC cell lines were assessed by flow cytometry after vehicle (DMSO, NT), g-PPT (20 μM), Gefitinib (100 nM) or g-PPT (20 μM) + Gefitinib (100 nM) treatment for 48 h. Lower panel, apoptotic rates were quantified. The results represent data from three independent experiments. p values were determined by one-way ANOVA. *p < 0.05, **p < 0.01, ***p < 0.001. (TIF 8614 kb)

Additional file 6: **Figure S5.** The expression of p-EGFR in murine tumors on conditional single or combine treatment with Gefitinib and g-PPT. Up panel, IF was used to detect the expression of p-EGFR in murine lung tumors with an anti-p-EGFR1086 antibody (red, left panel) and counterstaining with Hoechst (blue). For each experiment, five images of random fields were acquired. A representative image is shown (× 200/× 400 magnification). Down panel, the quantitation of p-EGFR, p values were determined by one-way ANOVA. *p < 0.05, **p < 0.01, ***p < 0.001. Data represent the mean ± SD of three replicate determinations. (TIF 10087 kb)

## Abbreviations

AKT: PKB (protein kinase B)
BMI: Body mass index
EGFR: Epidermal growth factor receptor
EGFR-TKI: Epidermal growth factor receptor tyrosine kinase inhibitors
ERK: Extracellular regulated protein kinases
FASN: Fatty acid synthase
g-PPT: 20s-Protopanaxatriol
LDs: Lipid droplets
MUFA: Saturated and monounsaturated fatty acids
NSCLC: Non-small cell lung cancer
OA: Oleic acid
OS: Overall survival
PFS: Progression free survival
PLIN: Perilipin
RECIST: Response evaluation criteria in solid tumors
SCD1: Stearoyl-CoA Desaturase 1
SFA: Saturated fatty acids
