# Supplementary material for: Co-administration of 20(S)-protopanaxatriol (g-PPT) and EGFR-TKI overcomes EGFR-TKI resistance by decreasing SCD1 induced lipid accumulation in non-small cell lung cancer
Source: J Exp Clin Cancer Res. 2019 Mar 15;38:129. doi: 10.1186/s13046-019-1120-4 (PMC6419820; doi:10.1186/s13046-019-1120-4)
Supplement: Supplementary file 1 — Table S1. Specimen type of patient collected. (DOCX 17 kb) [file 13046_2019_1120_MOESM1_ESM.docx]

Table S1: Specimen type of patient collected

| **Case No** | | **Gender** | Before TKI treatment | | **TKI treatment time**  **(month)** | After TKI treatment | |
| --- | --- | --- | --- | --- | --- | --- | --- |
|  |  |  | Paraffin-embedded tissue | Frozen tissue |  | Paraffin-embedded tissue | Frozen tissue |
| 01 | Female | | **+** | **-** | 12 | **+** | **-** |
| 02 | Female | | **+** | **+** | 9 | **+** | **+** |
| 03 | Female | | **+** | **+** | 7 | **+** | **+** |
| 04 | Male | | **+** | **-** | 7 | **+** | **-** |
| 05 | Male | | **+** | **-** | 3 | **+** | **-** |
| 06 | Male | | **+** | **-** | 3 | **+** | **-** |
| 07 | Female | | **+** | **+** | 2 | **+** | **+** |
| 08 | Female | | **-** | **-** | 5 | **+** | **+** |
| 09 | Male | | **-** | **-** | 3 | **+** | **+** |
| 10 | Male | | **-** | **-** | 3 | **+** | **+** |
| 11 | Male | | **+** | **+** | N | **-** | **-** |
| 12 | Male | | **+** | **+** | N | **-** | **-** |
| 13 | Female | | **+** | **+** | N | **-** | **-** |

Case number 01-07 patients after biopsy and EGFR mutational testing verified the presence of EGFR-TKI-sensitive mutations, under treatment with TKIs. Finally confirmed cTNM downstaging to IIIA at least and then the patients underwent initial surgery. Those patients harbor paired tissue of pre- and post- treatment.

Case number 07-10 patients were initially subjected to EGFR mutational testing using peripheral blood, tissue samples were collected only after TKI treatment. Case number 11-13 underwent initial surgery at the Department of Thoracic Surgery during the same period and were confirmed to possess sensitive EGFR mutations.

“+”means yes, have the type of tissue , “-”means no, without the type of tissue. Each specimen has a corresponding tumor and adjacent tissues.
