# Supplementary figures and images for: Co-administration of 20(S)-protopanaxatriol (g-PPT) and EGFR-TKI overcomes EGFR-TKI resistance by decreasing SCD1 induced lipid accumulation in non-small cell lung cancer
Source: J Exp Clin Cancer Res. 2019 Mar 15;38:129. doi: 10.1186/s13046-019-1120-4 (PMC6419820; doi:10.1186/s13046-019-1120-4)

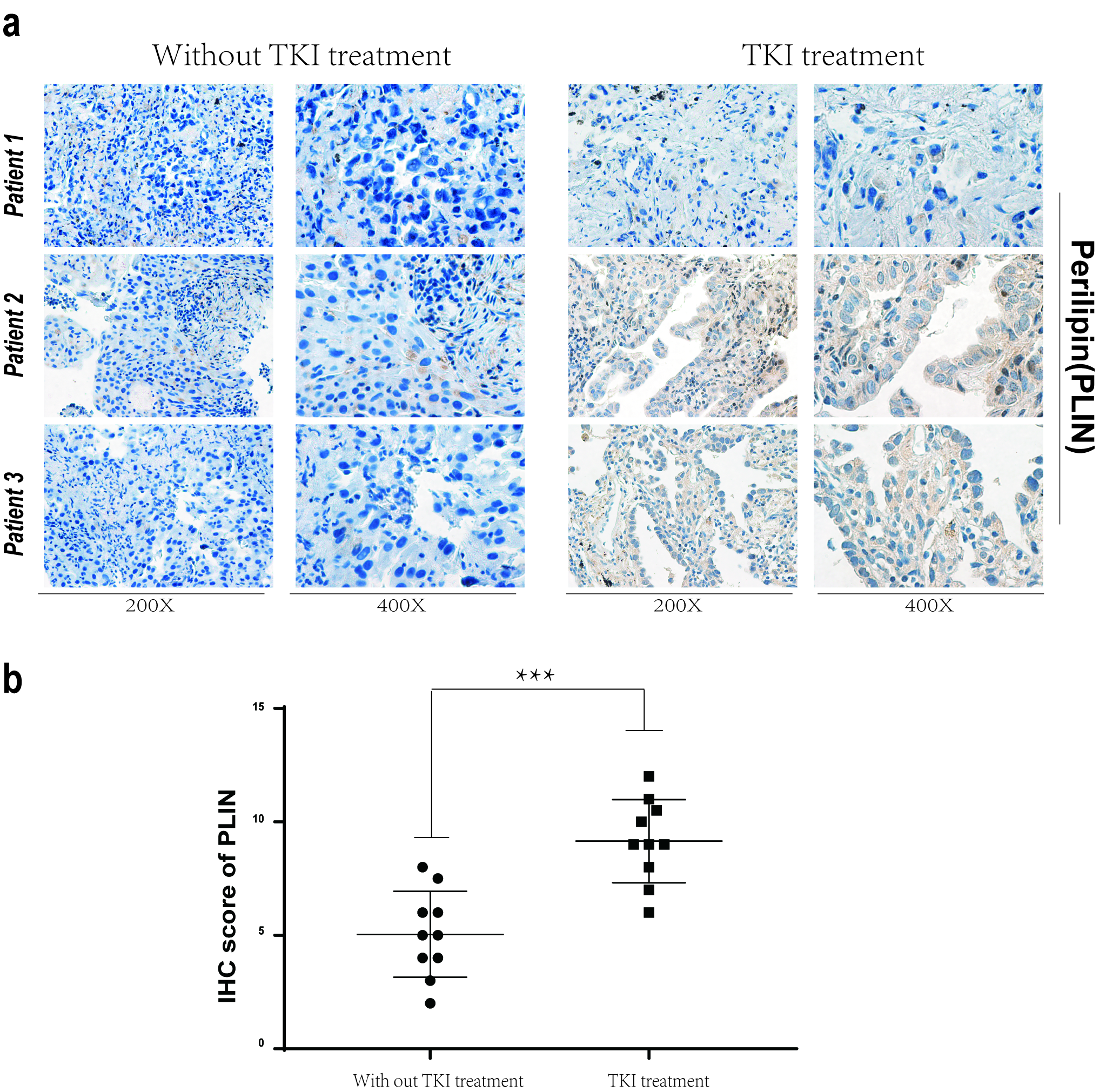

Supplement: Supplementary file 2 — Figure S1. The difference of PLIN expression between the pre- and post-Gefitinib treatment specimens (a) Immunohistochemical staining for perilipin proteins in 20 lung cancer tissues from 10 patients. The brown color in cancer cells denotes positive staining. Representative images of perilipin expression in NSCLC specimens from three representative patients are shown, including samples from both before and after EGFR-TKI treatment (× 200/× 400 magnification). (b) The immunohistochemical score for PLIN was significantly different between the pre- and post-Gefitinib treatment specimens. p values were determined by unpaired t test. ***p < 0.001. (TIF 10243 kb) [file 13046_2019_1120_MOESM2_ESM.tif]

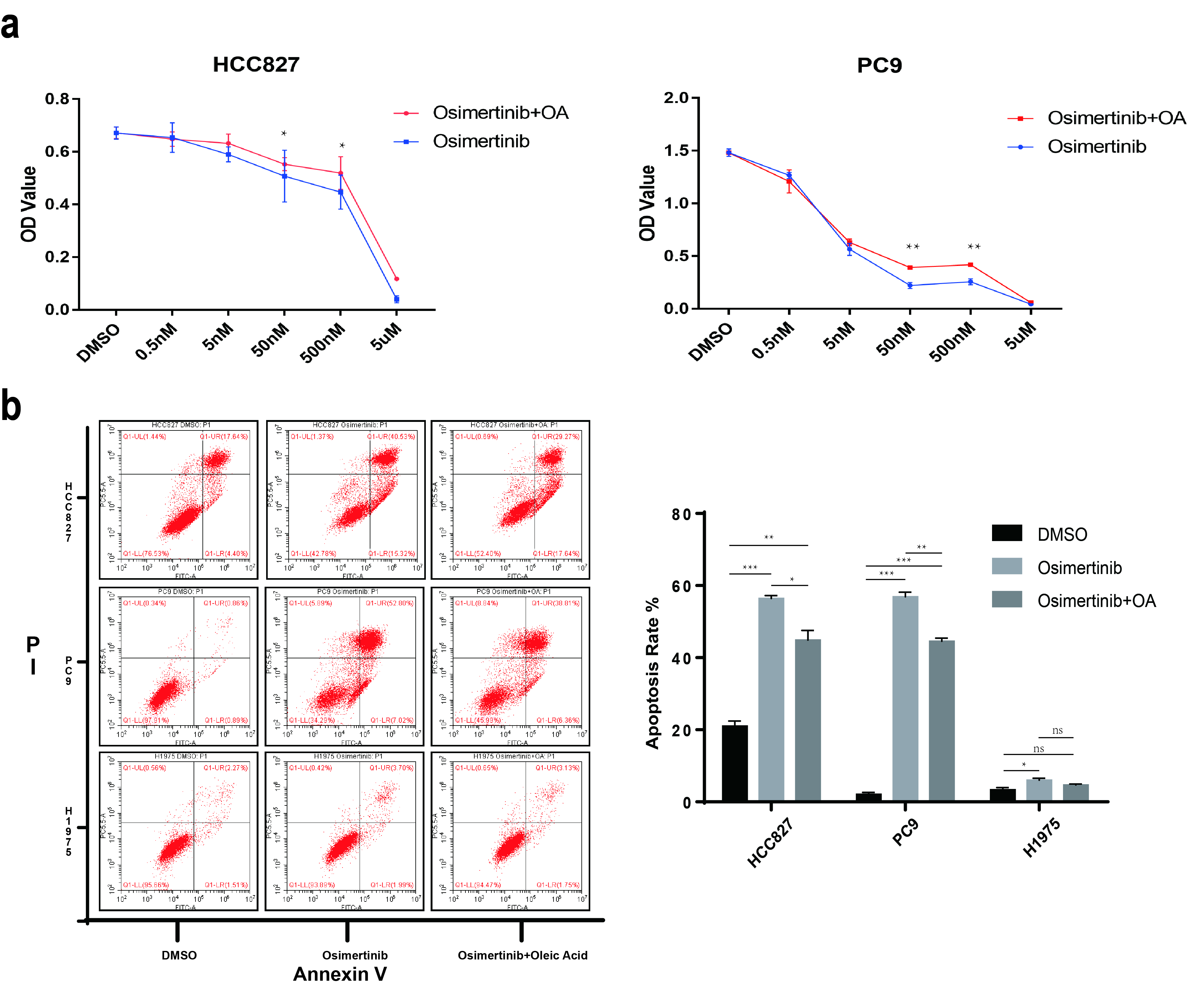

Supplement: Supplementary file 3 — Figure S2. OA abrogates the cytotoxic effect of osimertinib in EGFR sensitive mutation cell lines (a) The indicated NSCLC cell lines were exposed to various concentrations (0, 0.5 nM, 5 nM, 50 nM, 500 nM, and 5 μM) of osimertinib with vehicle (DMSO, NT) or OA (100 μM) for 2 days, and the relative OD value, representing the cell viability by CCK-8 assays, was assessed. Data represent the mean ± SD of three replicate determinations. (b) Upper panel, apoptotic rates of the indicated NSCLC cell lines were assessed by flow cytometry after vehicle (DMSO, NT), osimertinib (150 nM) or osimertinib (150 nM) + OA (100 μM) treatment for 48 h. Lower panel, apoptotic rates were quantified. The results represent data from three independent experiments. p values were determined by one-way ANOVA. *p < 0.05, **p < 0.01, ***p < 0.001. (TIF 3020 kb) [file 13046_2019_1120_MOESM3_ESM.tif]

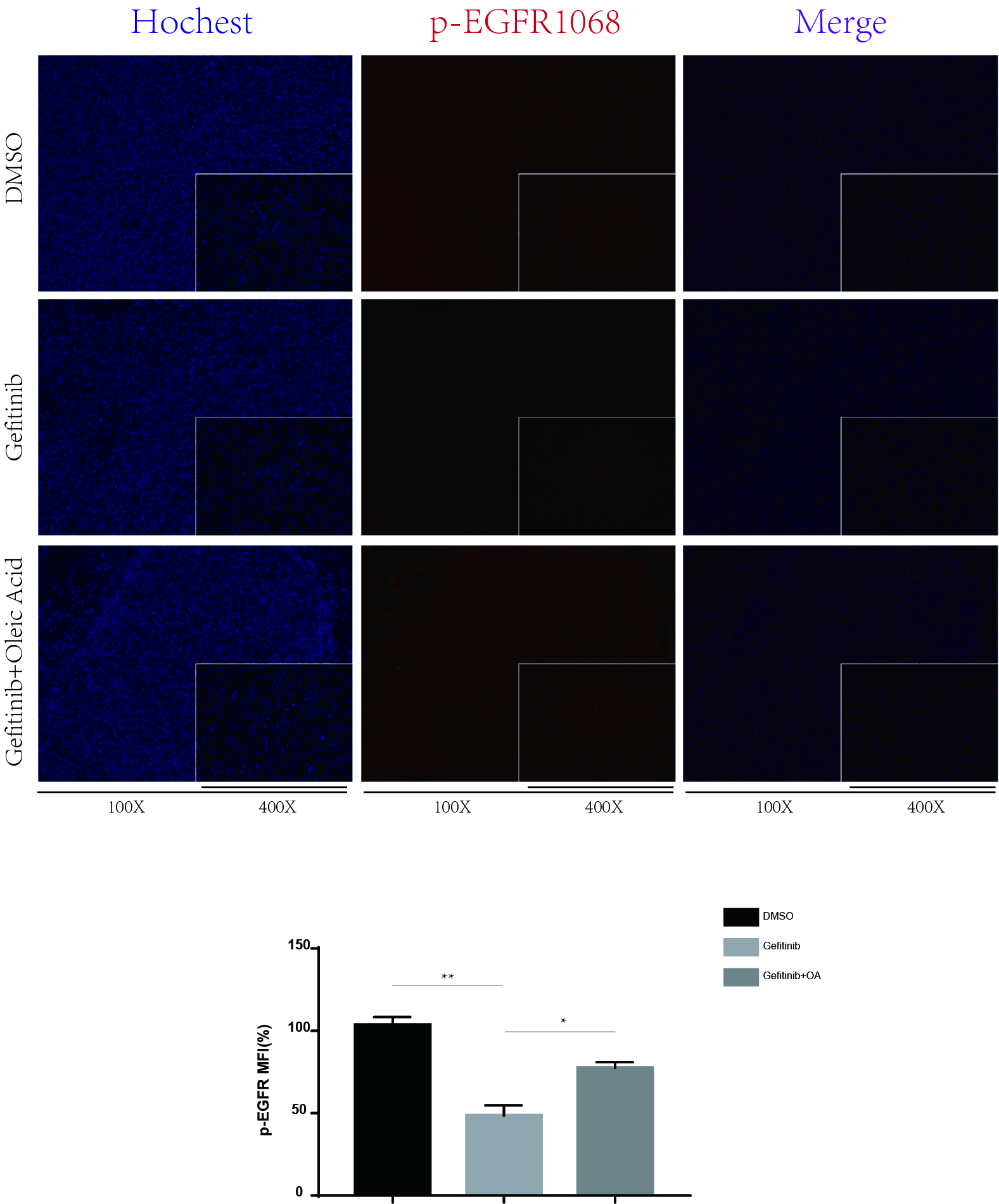

Supplement: Supplementary file 4 — Figure S3. When treatment with OA, induce the expression of p-EGFR in murine tumors Up panel, IF was used to detect the expression of p-EGFR in murine lung tumors with an anti-p-EGFR1086 antibody (red, left panel) and counterstaining with Hoechst (blue). For each experiment, five images of random fields were acquired. A representative image is shown (× 200/× 400 magnification). Down panel, the quantitation of p-EGFR, p values were determined by one-way ANOVA. *p < 0.05, **p < 0.01, ***p < 0.001. Data represent the mean ± SD of three replicate determinations. (TIF 9912 kb) [file 13046_2019_1120_MOESM4_ESM.tif]

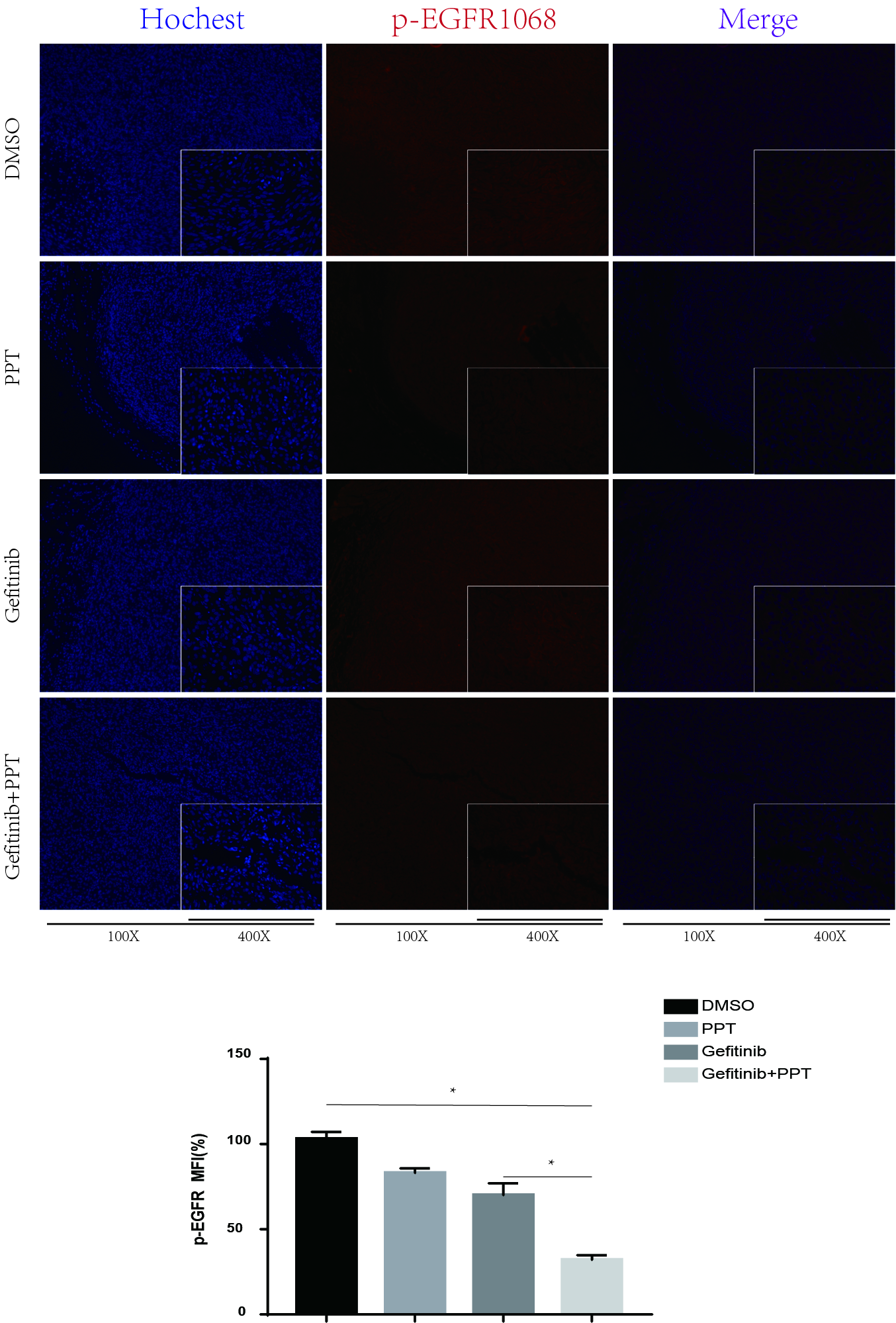

Supplement: Supplementary file 6 — Figure S5. The expression of p-EGFR in murine tumors on conditional single or combine treatment with Gefitinib and g-PPT. Up panel, IF was used to detect the expression of p-EGFR in murine lung tumors with an anti-p-EGFR1086 antibody (red, left panel) and counterstaining with Hoechst (blue). For each experiment, five images of random fields were acquired. A representative image is shown (× 200/× 400 magnification). Down panel, the quantitation of p-EGFR, p values were determined by one-way ANOVA. *p < 0.05, **p < 0.01, ***p < 0.001. Data represent the mean ± SD of three replicate determinations. (TIF 10087 kb) [file 13046_2019_1120_MOESM6_ESM.tif]
